# Ion- and water-binding sites inside an occluded hourglass pore of a trimeric intracellular cation (TRIC) channel

**DOI:** 10.1186/s12915-017-0372-8

**Published:** 2017-04-22

**Authors:** Xiaomin Ou, Jianli Guo, Longfei Wang, Hanting Yang, Xiuying Liu, Jianyuan Sun, Zhenfeng Liu

**Affiliations:** 1 National Laboratory of Biomacromolecules, CAS Center for Excellence in Biomacromolecules, Institute of Biophysics, Chinese Academy of Sciences, Beijing, China; 2 State Key Laboratory of Brain & Cognitive Sciences, CAS Center for Excellence in Biomacromolecules, Institute of Biophysics, Chinese Academy of Sciences, Beijing, China; 3 University of Chinese Academy of Sciences, Beijing, China

**Keywords:** Crystal structure, Ion channel, Membrane protein, Selectivity, Gating mechanism

## Abstract

**Background:**

Trimeric intracellular cation (TRIC) channels are crucial for Ca^2+^ handling in eukaryotes and are involved in K^+^ uptake in prokaryotes. Recent studies on the representative members of eukaryotic and prokaryotic TRIC channels demonstrated that they form homotrimeric units with the ion-conducting pores contained within each individual monomer.

**Results:**

Here we report detailed insights into the ion- and water-binding sites inside the pore of a TRIC channel from *Sulfolobus solfataricus* (*Ss*TRIC). Like the mammalian TRIC channels, *Ss*TRIC is permeable to both K^+^ and Na^+^ with a slight preference for K^+^, and is nearly impermeable to Ca^2+^, Mg^2+^, or Cl^–^. In the 2.2-Å resolution K^+^-bound structure of *Ss*TRIC, ion/water densities have been well resolved inside the pore. At the central region, a filter-like structure is shaped by the kinks on the second and fifth transmembrane helices and two nearby phenylalanine residues. Below the filter, the cytoplasmic vestibule is occluded by a plug-like motif attached to an array of pore-lining charged residues.

**Conclusions:**

The asymmetric filter-like structure at the pore center of *Ss*TRIC might serve as the basis for the channel to bind and select monovalent cations. A Velcro-like plug-pore interacting model has been proposed and suggests a unified framework accounting for the gating mechanisms of prokaryotic and eukaryotic TRIC channels.

**Electronic supplementary material:**

The online version of this article (doi:10.1186/s12915-017-0372-8) contains supplementary material, which is available to authorized users.

## Background

The regulated processes of Ca^2+^ release from the intracellular stores and its uptake from the cytosol are vital for various biological processes including muscle contraction, neurotransmitter release, cell division, and apoptosis [1, 2]. For instance, muscle contraction is initiated by membrane depolarization followed by opening of a ryanodine receptor (RyR) channel to release Ca^2+^ from the lumen of the sarcoplasmic reticulum (SR) into the cytosol. The process is known as excitation-contraction (E-C) coupling [3]. Rapid efflux of Ca^2+^ from the SR generates a transient negative potential inside the SR lumen and will hinder Ca^2+^ release if the transmembrane potential remains unbalanced. Thus, efficient operation of E-C coupling requires not only the RyR to release Ca^2+^, but also counteracting ion channels to restore the balance of the SR membrane potential and maintain ion homeostasis within the SR lumen [4].

Two isoforms of SR/endoplasmic reticulum (ER) membrane proteins, called trimeric intracellular cation (TRIC) channels (TRIC-A and TRIC-B), presumably function as the counteracting ion channels facilitating the intracellular Ca^2+^ handling processes [5]. Alternatively, they might serve to restore the balance of trans-SR K^+^ after the RyRs close, instead of carrying countercurrent during Ca^2+^ release [6]. TRIC channels are permeable to K^+^ and Na^+^ with moderate selectivity for K^+^ over Na^+^ and are impermeable to Ca^2+^, Mg^2+^, or anions [5, 7]. TRIC-A and TRIC-B have distinct single-channel conductances as well as diverse regulatory mechanisms and physiological roles [8, 9]. TRIC-A is regulated by transmembrane voltage [8] and may interact with the RyR functionally and physically [9, 10]. TRIC-B channel is activated by micromolar Ca^2+^ applied on the cytosolic side, but it is inhibited by Ca^2+^ on the luminal side [8] and is also regulated by voltage [11]. TRIC-B may modulate the Ca^2+^-release channel activity of inositol trisphosphate (IP_3_) receptor [9] and is essential for perinatal lung maturation [12]. Genetic mutations of the human *TMEM38B* gene (encoding the TRIC-B protein) are found in patients with a hereditary brittle bone disease called osteogenesis imperfecta [13–16]. *TMEM38B* knockout mice are deficient in producing collagen, and bone mineralization is impaired in the mutant animals [17, 18]. Moreover, TRIC-A contributes to the maintenance of normal blood pressure and may serve as a potential pharmaceutical target for treating hypertension [19, 20]. The association of TRIC channels with bone, pulmonary, and muscular diseases indicates that they have indispensable functions in the related physiological and developmental processes [9, 17, 18, 21].

The structures of TRIC-B channels from *Caenorhabditis elegans* (*Ce*TRIC-B1 and *Ce*TRIC-B2) revealed a homotrimeric membrane protein-lipid complex with hourglass-shaped pores traversing through each individual monomer [22]. A phosphatidylinositol 4,5-biphosphate (PtdIns(4,5)P_2_, also known as PIP_2_) lipid molecule binds specifically to each monomer at 1:1 stoichiometry, mediates trimerization of *Ce*TRIC-B channels, and stabilizes the cytoplasmic gate of the channel. In addition to the members found in eukaryotic organisms, TRIC channel orthologs are widespread in prokaryotes including bacteria and archaea [23]. The prokaryotic TRIC members form the largest group of the TRIC family, outnumbering eukaryotic ones. A recent study suggested that they may mediate K^+^ uptake, and the structures of two prokaryotic TRIC orthologs from *Rhodobacter sphaeroides* (*Rs*TRIC) and *Sulfolobus solfataricus* (*Ss*TRIC) were reported at 3.4- and 2.6-Å resolution, respectively [24]. These prokaryotic orthologs form homotrimeric units resembling those of *Ce*TRIC-B channels, and the ion-conducting pore is also contained within each individual monomer [24].

Although significant progress has been made on structural and functional studies of both eukaryotic and prokaryotic TRIC channels, the fundamental mechanistic questions regarding the molecular basis of ion selectivity remain largely open. The pore architectures of TRIC channels clearly do not resemble those of homotetrameric K^+^ channels, such as the well-studied KcsA channel [25]. We do not know where the ion selectivity filter is located in TRIC channels or how TRIC channels selectively bind monovalent cations in their pore region. Therefore, it is indispensable to characterize the ion-binding sites along the permeation pathway of TRIC channels through high-resolution structural studies of TRIC channels. Moreover, the lack of the PIP_2_ molecule in prokaryotic TRIC orthologs raises questions about how their gates are stabilized in the absence of PIP_2_ in their structure. Here we describe the detailed ion- and water-binding sites inside the pore of each *Ss*TRIC monomer, define the chemical basis of K^+^ coordination inside a buried asymmetric filter-like structure, and provide new insights into a Velcro-like plug-pore interacting model accounting for the gating mechanism of TRIC channels.

In the following Results and Discussion sections, Additional files 1, 2, 3, 4, 5, 6, 7, 8, 9, 10 provide supplemental information. Additional file 11 provides supporting data for Figs. 2 and 5.

## Results

### Ion- and water-binding sites within an hourglass-shaped pore

**Fig. 1:**
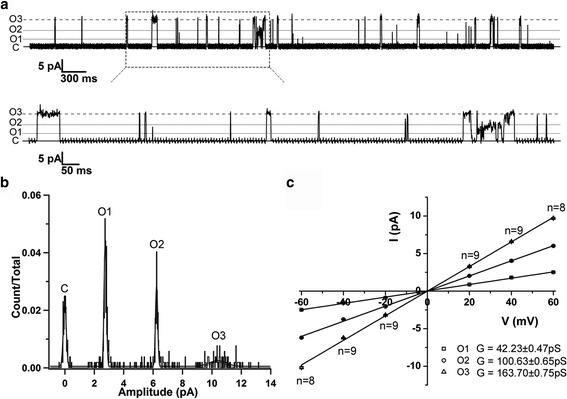
Electrophysiological recording of the activities of the *Ss*TRIC channel reconstituted on liposomes. **a** Representative recordings of the wild-type *Ss*TRIC measured at +60 mV. Three major open states are observed in this patch, presumably attributed to the opening of one (*O1*), two (*O2*), and three (*O3*) monomeric pores within an *Ss*TRIC trimer. **b** The all-points amplitude histogram of the electrophysiological data of the wild-type *Ss*TRIC channel recorded at +60 mV. The bin width is set at 0.03 pA/bin. The histograms were fitted with four Gaussian functions. The four peaks correspond to the closed state (*C*), one monomer open (*O1*), two monomers open (*O2*), and three monomers open (*O3*). **c** The current-voltage relationships of the *Ss*TRIC channel at O1, O2, and O3 states. The error bars indicate the standard errors of mean values (*SEM*). The number of measurements (*n*) is labeled above or below the data points

**Fig. 2:**
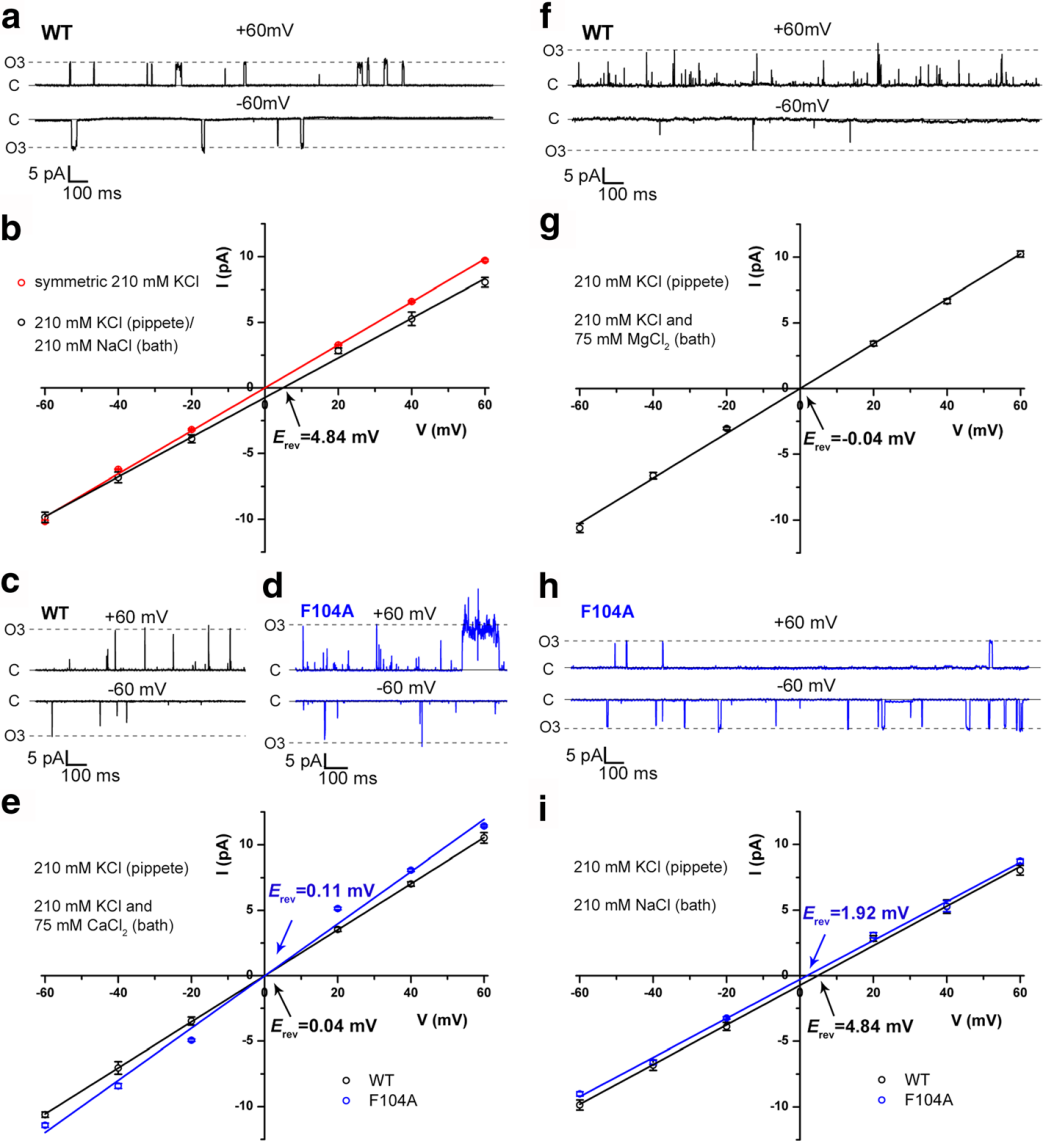
Ion selectivity of *Ss*TRIC channel. **a** and **b** Representative recordings and current-voltage relationships of the wild-type *Ss*TRIC measured under bi-ionic condition of 210 mM KCl (pipette) and 210 mM NaCl (bath). The I/V curve of wild-type *Ss*TRIC measured under symmetrical 210 mM KCl (*red*) is included for comparison. The reversal potential (4.84 ± 0.86 mV for bi-ionic condition and −0.02 ± 0.63 mV for symmetrical condition) is derived by linear fitting of the I/V relationship data. The error bars indicate the standard errors of mean values (*SEM*, number of patches/*n* = 5 for bi-ionic condition, and *n* = 8 or 9 for symmetrical condition as shown in Fig. 1c). **c**, **d**, and **e** Representative recordings and current-voltage relationships of the wild-type *Ss*TRIC (*dark*) and F104 A mutant (*blue*) measured under bi-ionic condition of 210 mM KCl (pipette)/210 mM KCl + 75 mM CaCl_2_ (bath). The reversal potential is at 0.04 ± 0.09 mV (mean ± SEM, *n* = 4 except that *n* = 3 for the data point at −20 mV) for the wild type and 0.11 ± 1.77 mV (mean ± SEM, *n* = 5) for the F104A mutant. **f** and **g** Representative recordings and current-voltage relationships of the wild-type *Ss*TRIC measured under bi-ionic condition of 210 mM KCl (pipette)/210 mM KCl + 75 mM MgCl_2_ (bath). The reversal potential is at −0.04 ± 0.69 mV (mean ± SEM, *n* = 3). **h** and **i** Representative recordings and current-voltage relationships of F104A mutant measured under bi-ionic condition of 210 mM KCl (pipette)/210 mM NaCl (bath). For comparison, the I/V curve of wild-type t*Ss*TRIC under the same condition is included **i** The reversal potential is at 1.92 ± 0.93 mV (mean ± SEM, *n* = 5 except that *n* = 3 for −20 mV and *n* = 4 for 40 mV) for F104A mutant or 4.84 ± 0.86 mV (mean ± SEM, *n* = 5) for the wild type. See Additional file 11 for the supporting data values

As shown in Fig. 1, the *Ss*TRIC protein reconstituted on giant unilamellar vesicles (GUVs) forms an ion channel permeable to K^+^ ion and exhibits three major open states with conductances at 42.2 pS, 100.6 pS, and 163.7 pS, respectively. These conductances are similar to those of the *Ce*TRIC-B1 channel (45.8 pS, 101.5 pS, and 153.2 pS for the first, second, and third open states, respectively) reported previously [22], suggesting that their open pores may have similar sizes. Furthermore, the *Ss*TRIC channel is also permeable to Na^+^ with a *P*_K_/*P*_Na_ ratio at 1.21 (derived from the reversal potential at 4.84 mV under the 210 mM KCl/210 mM NaCl bi-ionic condition; see Fig. 2a, b), close to the *P*_K_/*P*_Na_ value (1.5) of the mammalian TRIC-A channel reported previously [5]. Meanwhile, *Ss*TRIC is essentially impermeable to Ca^2+^, Mg^2+^, or Cl^–^, as the reversal potential remains close to 0 mV when 75 mM CaCl_2_ or MgCl_2_ is added to the bath solution (the KCl concentration is the same on both sides; see Fig. 2c, e–g).

**Fig. 3:**
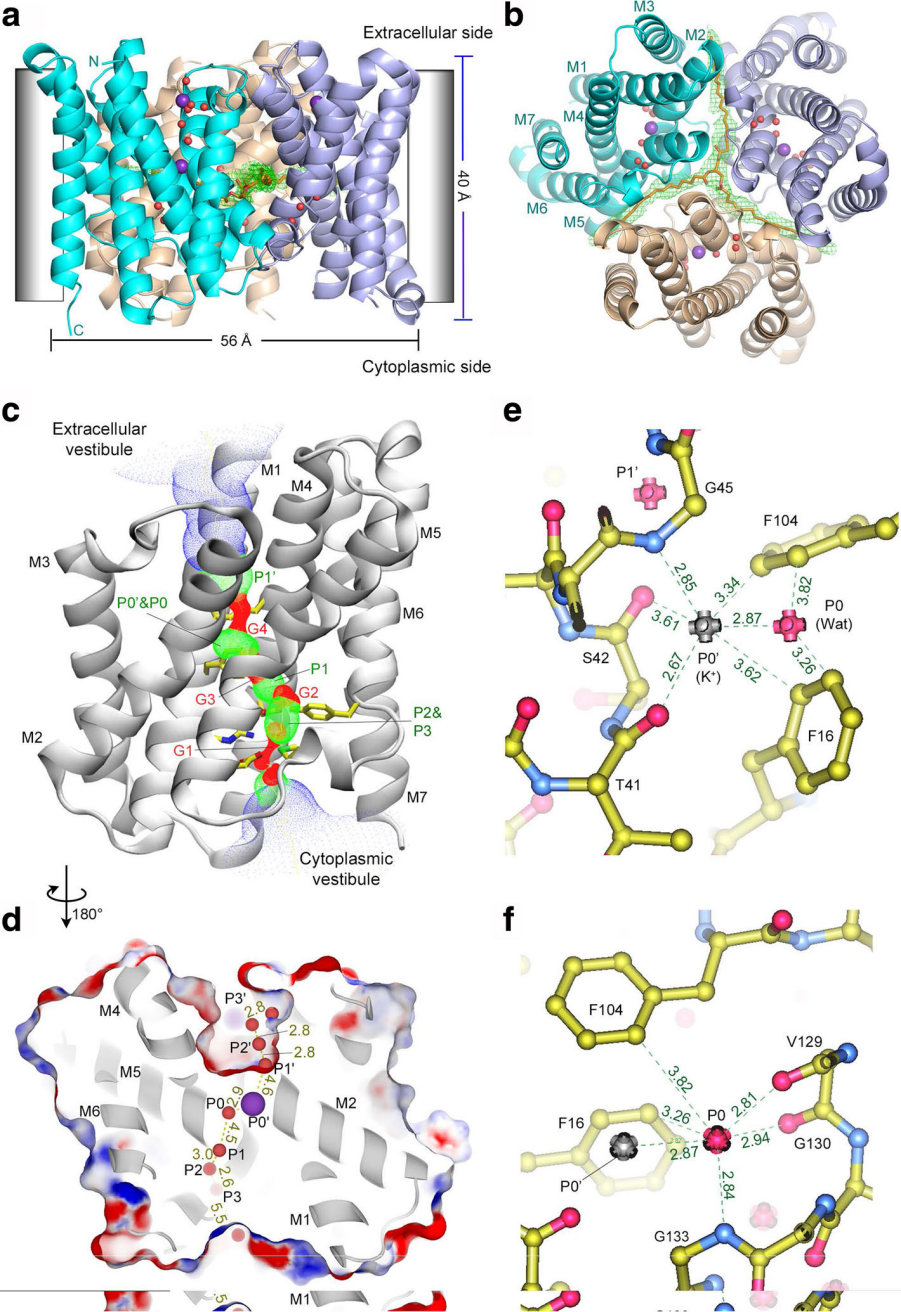
Ion- and water-binding sites within the pores of *Ss*TRIC channel. **a** Structure of a *Ss*TRIC homotrimer at 2.2-Å resolution viewed along the membrane plane. The estimated position of membrane is indicated by the *light gray boxes*. The K^+^ ions and water molecules within the pore region are shown as *purple* and *red* spheres, respectively. **b** The *Ss*TRIC trimer viewed along the C3 axis from the cytoplasmic side. The *green meshes* indicate the 2*F*_o_-*F*_c_ electron density (contoured at +1.0 × σ level) of a neutral lipid molecule. **c** The pore running through *Ss*TRIC monomer is indicated by the *tri-color dotted areas. Red areas*, radius <1.15 Å; *green areas*, 1.15 Å < radius < 2.30 Å; *blue areas*, radius >2.30 Å. G1–G4 label the pore constriction sites (or gates) as shown by the red areas, while the green areas contain the binding sites for ion or water molecules labeled as P1′, P0′, P0, P1–P3 in **d**. The key amino acid residues lining the pore lumen are shown as stick models. From middle to bottom, they are F104, F16, Y153, R137, D138, and M146. **d** Distribution of K^+^ ions or water molecules within the pore region of an *Ss*TRIC monomer. The sectional view of a monomer is shown as the electrostatic potential surface model overlaid with the cartoon model (color code: *deep red*, electronegative; *deep blue*, electropositive; *silver*, cartoon model of the protein backbone). The distances between two adjacent molecules are shown by the *numbers* (Å) labeled around the *dashed lines*. **e** A zoom-in view of the pore center with two ion-/water-binding sites. K^+^ ions and water molecules are shown as *silver* and *red bullets*, respectively. Oxygen, nitrogen, and carbon atoms of the amino acid residues are colored *red, blue*, and *yellow*, respectively. The interactions between K^+^ ion on P0′ site (or water molecule on P0 site) and the surrounding residues are indicated by the *dashed lines* with their distances (Å) labeled nearby. **f** The pore center viewed from a different angle to show the water molecule on P0 site and its surrounding residues

The structure of *Ss*TRIC solved at 2.2-Å resolution shows that it forms a symmetrical homotrimer measuring ~56 Å wide and ~40 Å tall, and has flat surfaces on both the extracellular and cytoplasmic sides (Fig. 3a and b). It superposes well with the previous structures of *Ss*TRIC in complex with the Fab fragment of a monoclonal antibody (*Ss*TRIC-Fab complex, root mean square deviation (RMSD) of C_α_ atoms = 0.39 Å), and a bacterial TRIC homolog (*Rs*TRIC) from *Rhodobacter sphaeroides* [24] (RMSD of C_α_ atoms = 2.36 Å) (see Additional file 1: Figure S1). When compared to *Ce*TRIC-B1 or the RyR (both are membrane proteins located on the ER/SR membrane), the membrane-embedded domain of the *Ss*TRIC trimer appears to be ~6 Å taller along the dimension parallel to the membrane normal (Additional file 2: Figure S2). Curiously, the tilt angles of transmembrane helices in *Ss*TRIC with respect to the membrane normal are dramatically different from those in C*e*TRIC-B1/B2 (Table 1). Except for M6, most transmembrane helices (M1–M5 and M7) in *Ss*TRIC are tilted from the membrane normal at smaller angles compared to those in *Ce*TRIC-B1/B2. This may arise from the adaptation of membrane proteins to different membrane environments, i.e., the plasma membrane of prokaryotic cells versus ER membranes of eukaryotic cells.

**Table 1:** The tilt angles of transmembrane helices in *Ss*TRIC and *Ce*TRIC-B1/B2

Transmembrane helices		η angle (°)	
	*Ss*TRIC	*Ce*TRIC-B1	*Ce*TRIC-B2
M1	26.9	31.5	34.8
M2a	46.0	64.1	66.9
M2b	41.5	63.0	61.7
M3	14.1	21.2	20.7
M4	19.0	46.7	46.2
M5a	51.8	54.6	55.6
M5b	37.8	59.4	60.4
M6	25.3	17.7	20.3
M7	4.7	13.2	19.9

The tilt angle (η) is defined as the angle between the axis of each individual transmembrane helix and the central C3 axis (parallel with the membrane normal) of the *Ss*TRIC trimer or *Ce*TRIC-B1/B2 trimer

Both eukaryotic and prokaryotic TRIC channels harbor their ion-conducting pores within each subunit of the homotrimeric assemblies [22, 24]. As shown in Fig. 3c, an hourglass-shaped pore runs through each *Ss*TRIC monomer from cytoplasm to extracellular space. Along the pore, four bottleneck sites, including two (G1 and G2) on the cytoplasmic side, one at the central region (G3), and one close to the extracellular vestibule (G4), form highly constricted areas with extremely small widths at 0.7–2.0 Å (smaller than the diameter of a K^+^ ion at ~2.8 Å [26]). For the pore in *Rs*TRIC, the G4 site around Phe103 appears to be slightly wider than the one in *Ss*TRIC, although their overall profiles and pore-lining residues are very similar (Additional file 3: Figure S3a and b). For comparison, the pore within each *Ce*TRIC-B monomer harbors two bottlenecks (instead of four), one on the cytoplasmic side and the other on the luminal side of the ER/SR [22] (Additional file 3: Figure S3c). The pore lumen surface of *Ss*TRIC is mainly shaped by amino acid residues from M1, M2, M4, and M5 helices. Within the pore, a single file of eight well-resolved spherical densities was observed in the high-resolution electron density map of the K^+^-soaked crystal (Additional file 4: Figure S4a and b). The four ion/water molecules (P0–P3) on the cytoplasmic side are related to the other four at the P0′–P3′ sites on the extracellular side, and they approximately follow the internal pseudo-C_2_ symmetry [22, 24] between the M1-M2-M3 and M4-M5-M6 helices. Among these densities, two (P0 and P0′) are located at the pore center area between the G3 and G4 bottleneck sites (Fig. 3c), and they intercalate in the grooves of glycine-rich kinks around Ala44-Gly46 and Gly130-Gly134 regions on M2 and M5, respectively (Fig. 3e and f). Previously, two spherical *F*_o_-*F*_c_ densities at positions similar to P0 and P0′ were observed in the structure of the *Ss*TRIC-Fab complex and assigned as water molecules [24].

To test whether any of these potential ion-/water-binding sites inside the pore can be accessed by monovalent cations, we have soaked the *Ss*TRIC crystal with Tl^+^, the electron-rich surrogate of the K^+^ ion [27]. The anomalous difference Fourier peaks of Tl^+^ ions are mostly detected on the P0′ and P3′ (outer) sites (Tl-1 and Tl-2 shown in Additional file 4: Figure S4c) inside the pore (and on numerous surface sites), but not on the P0–P3 (inner) sites. By referring to the positions of the Tl^+^ peaks, the putative K^+^ binding sites have been assigned in the structure of *Ss*TRIC soaked with KCl. As shown in Fig. 3e and f, the K^+^ ion bound on the P0′ site is mainly coordinated by the backbone carbonyl from Thr41, backbone amide from Gly45, and a water molecule nearby on the P0 site. Such a coordination mode resembles one half of the K^+^ coordination observed previously in valinomycin (a K^+^-permeable ionophore, see Additional file 5: Figure S5a and b), but differs from the square antiprism-type coordination in KcsA (Additional file 5: Figure S5a and c). Due to steric hindrance of the bulky side chain of Phe104, three additional ligands below K^+^ (as in valinomycin) are absent in *Ss*TRIC. The water molecule on the P0 site is further ligated to the backbone carbonyl groups of Val129 and Gly130 and the backbone amide of Gly133. The coordination bond lengths between K^+^ and its ligands on P0′ site are consistent with those (2.7–2.9 Å) observed in the KcsA channels, although the coordination geometry of K^+^ in *Ss*TRIC is of trigonal shape instead of the square antiprism found in KcsA [25]. The introduction of a G45A/G47A or G132A/G133A mutation in *Rs*TRIC (corresponding to A44/G46 and G133/134 in *Ss*TRIC) abolishes its channel activity [24], indicating that the kinks on M2 and M5 helices are essential for the function of TRIC channels. In these two mutants, the kinks may have been distorted, leading to loss of the P0′ or P0 site (both crucial for K^+^ binding). Similar kinks are also present on the M2 and M5 helices of *Ce*TRIC-B channels, and a Rb^+^ ion was found on a site nearby these regions [22]. Thus, such a double-kink structure with an inverted twofold pseudo-symmetry may represent a general characteristic feature of the TRIC channel family.

In *Ss*TRIC, the aromatic rings of Phe16 from the M1 helix and Phe104 from the M4 helix enclose the P0 and P0′ sites from the other side opposing to the M5 kink and M2 kink, respectively (Fig. 3e and f). The side chains of Phe104 and Phe16 not only serve to constrict the pore center to form two bottlenecks (G3 and G4 in Fig. 3c) but may also contribute to binding K^+^ through cation-π interactions [28]. Mutation of Phe104 to alanine decreases the *P*_K_/*P*_Na_ ratio from 1.21 to 1.08 (as the reversal potential shifts from 4.84 to 1.92 mV under KCl/NaCl bi-ionic conditions, see Fig. 2h and i), and the F104A mutant is also nearly impermeable to Ca^2+^ or Cl^–^ as the wild type (Fig. 2d and e). Therefore, Phe104 may have a crucial role in adjusting the permeability of *Ss*TRIC to K^+^ versus Na^+^, presumably by shaping the geometry of the P0′ site slightly more favorably for binding K^+^ than Na^+^. Corresponding to the two phenylalanine residues in *Ss*TRIC, *Ce*TRICB1/B2 channels have their His34/35 and Lys136/Lys137 positioned nearby the kinks to enclose their central ion-binding sites [22]. Such a putative asymmetric cation-binding site at the pore center likely serves as the basis for TRIC channels to selectively bind and conduct monovalent cations (K^+^ and Na^+^) when the flanking bottleneck sites become wider at the open state. Besides the K^+^/Tl^+^ ion on the P0′ site, the one on the P3′ site in the extracellular vestibule of the pore is coordinated by the side chain carboxyl of Asp50 and the backbone carbonyl of Gly46. This peripheral site is close to the extracellular surface, and it may serve to attract K^+^ ions before they reach the pore center, or facilitate the release of K^+^ to the extracellular space.

### Gain-of-function mutants

To further verify whether the ion permeation pathway of the *Ss*TRIC channel does traverse through each monomer as observed in the structure, we have introduced a series of alanine mutations to the key amino acid residues lining the pore lumen surface (Additional file 6: Figure S6a). Expression of the wild-type *Ss*TRIC channel in *Escherichia coli* induces moderate inhibition of cell growth, while the empty vector or expression of a different membrane protein (the large-conductance mechanosensitive channel from *E. coli*, *Ec*MscL) has little effect on cell growth (Additional file 6: Figure S6b and c). Such an inhibitory effect is likely due to the basal leaky activity of the *Ss*TRIC channel stimulated by the resting membrane potential (Δ*ψ*) of *E. coli* cells. The activated *Ss*TRIC channel may leak out intracellular K^+^ ions, perturb the resting potential balance, and inhibit the growth of *E. coli* cells moderately (Additional file 6: Figure S6b). Mutation of the key residues along the pore lumen surface (such as F104A, D97A, R137A, D138A, M146A, and Y153A) leads to gain-of-function (GOF) phenotypes compared to the wild-type channel (Additional file 6: Figure S6b), resembling the phenotype of a well-established severe GOF mutant (G26H) of MscL [29, 30]. Unlike the other mutants, the F16A protein is undetectable in the membrane fraction (Additional file 6: Figure S6d) or at the whole-cell level, indicating that the protein may have been degraded before forming a functional channel on the membrane due to its high toxicity. As a result, the cells hosting the F16A mutant (not expressing) grow nearly as normal as the empty vector. The control, the R187A mutant (at a site distant from the pore region), expresses normally and shows phenotypes similar to the wild type (Additional file 6: Figure S6c). Hence, the in vivo functional assay data are consistent with the observation of a potential ion permeation pathway contained within each *Ss*TRIC monomer.

### Neutral lipid instead of PIP_2_ bound to *Ss*TRIC

At the monomer-monomer interfaces of the *Ss*TRIC trimer, lipid-like moieties have been observed (Fig. 3a and b), but the identities of these interfacial cofactors are elusive [24]. They have elongated tubular features extending from the peripheral region toward the center of the *Ss*TRIC trimer, and three fatty acyl group-like chains join at the center to form a triskelion-shaped structure enhancing the association of three *Ss*TRIC monomers through hydrophobic interactions. Thin layer chromatography and mass spectrometry analyses of the lipid samples extracted from purified *Ss*TRIC preparations suggest that they belong to highly hydrophobic neutral lipid species (likely a mixture of triacylglycerol and free fatty acids/derivatives from the *E. coli* membrane, Additional file 7: Figure S7a–c). A similar pattern of lipid bands is also observed in the sample extracted from the pure preparation of TRIC ortholog from *E. coli* (*Ec*TRIC, Additional file 7: Figure S7a). These lipid molecules are not random species bound to *Ss*TRIC. Instead, they are most likely specific lipids selected by the hydrophobic binding site with a well-defined triskelion shape. Electrospray ionization mass spectrometry analysis detected the presence of a molecule with an m/z value of 987.7, likely corresponding to an ionized triacylglycerol molecule, in the lipid samples extracted from purified *Ss*TRIC protein preparation (Additional file 7: Figure S7b and c). Therefore, the triskelion-shaped density in the *Ss*TRIC trimer is interpreted as a triacylglycerol molecule, and the model fits reasonably well with the electron density (Additional file 7: Figure S7d and e). The amino acid residues involved in binding the lipid are mainly hydrophobic residues from the M2 and M5 helices (Additional file 7: Figure S7e and f). In comparison, *Ce*TRIC-B channels contain a PIP_2_ lipid molecule per monomer, and the two fatty acyl chains of the PIP_2_ molecule extend to the monomer-monomer interfaces (Additional file 7: Figure S7g and h), both contributing to trimerization simultaneously [22]. Like the lipid in *Ss*TRIC, the fatty acyl chains of PIP_2_ molecules in *Ce*TRIC-B channels are also surrounded by hydrophobic residues from the M2 and M5 helices (Additional file 7: Figure S7h and i). Nevertheless, the bulky hydrophilic inositol 4,5-biphosphate head group (covalently linked to the 3-position of the glycerol backbone) of PIP_2_ inserts through the gap between the M5 and M6 helices, and is wrapped at the center of each monomer to stabilize the cytoplasmic gate of *Ce*TRIC-B channels (Additional file 7: Figure S7g and h). For the lipid molecule in *Ss*TRIC, the three fatty acyl groups (instead of two) extend laterally through the fenestration between M6 and M2′ (from adjacent monomer) helices. Instead of bending toward the pore region within each monomer (like the PIP_2_ head group), they have reached the external surface facing lipid bilayer (Additional file 7: Figure S7d and e). The two positively charged residues (Lys130 and Arg134 in *Ce*TRIC-B2 shown in Additional file 7: Figure S7h) involved in binding the PIP_2_ head group in *Ce*TRIC-B are absent in *Ss*TRIC or other prokaryotic orthologs. No bulky hydrophilic head groups are attached to the fatty acyl chains of the lipid found in the *Ss*TRIC channel, suggesting that prokaryotic TRIC channels may adopt a different mechanism to stabilize the cytoplasmic gate. It is noteworthy that *Sulfolobus solfataricus* and other archaea contain ether-linked lipids (such as *sn*-2,3-diphytanylglycerol diether and glycerol-dialkyl-calditol-tetraether) [31] instead of the ester-linked lipids commonly found in bacterial or eukaryotic cells. Therefore, the *Ss*TRIC from its native cellular environments may adopt the ether lipids in its structure.

### A tethered plug-like motif at the cytoplasmic gate

**Fig. 4:**
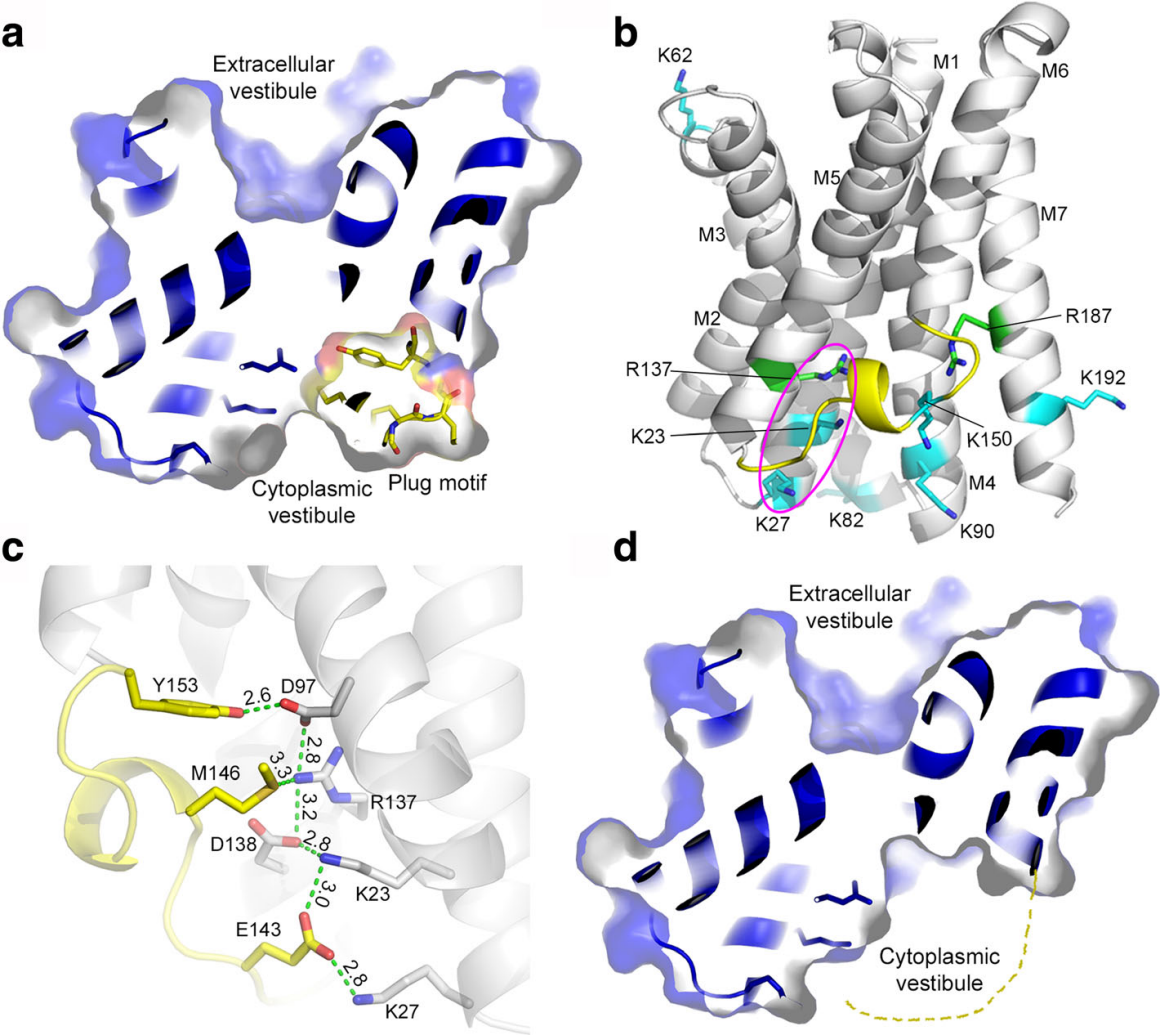
A tethered plug motif in *Ss*TRIC channel. **a** Occlusion of the cytoplasmic vestibule by the plug motif. The sectional view of the surface model of *Ss*TRIC structure shows the position of plug motif inside the cytoplasmic vestibule. **b** The plug motif tethered in the loop region between M5 and M6 helices. The plug motif is highlighted in *yellow*, and the basic residues are shown as stick models colored in *cyan* (Lys) or *green* (Arg). The *magenta elliptical rings* define an array of three basic residues nearby the plug motif. **c** The Velcro-like interactions between the plug motif and the potential voltage-sensing basic residues. *Green dashed lines* indicate the hydrogen bonds or ionic interactions between two adjacent residues. Bond lengths (Å) are labeled nearby the dashed lines. **d** A model of *Ss*TRIC with plug motif dislocated or removed. For comparison, the view is identical to the one shown in a. The cytoplasmic vestibule appears much wider and extends closer toward the bottom of extracellular vestibule. The *dashed line* denotes the mobilized plug motif at an unknown position

How does *Ss*TRIC stabilize its cytoplasmic gate in the absence of a bulky lipid head group? The pore of the *Ss*TRIC channel is more open on the extracellular side than the intracellular side (Fig. 4a). What is the cause of such dramatic differences between the outer and inner vestibules of the *Ss*TRIC channel? Each *Ss*TRIC monomer consists of three motifs, called motif A, motif B, and motif C, respectively (Additional file 8: Figure S8). Motif A contains M1, M2, M3, and two short amphipathic helices. Motif B comprises M4, M5, M6, and one short amphipathic helix in the M5–M6 loop region. These two motifs correspond to the N-repeat and C-repeat described in the previous study [24]. Motif A is related to motif B by a pseudo-C2 axis running approximately parallel to the membrane plane (Additional file 8: Figure S8a and b). They superpose well with the root mean square deviation (RMSD) of α-carbon atoms at 1.42 Å (Additional file 8: Figure S8b), and their sequences share 25% identity (Additional file 8: Figure S8c). The side chains of three key residues from motif B, namely Tyr153, Met146, and Arg137, collectively contribute to occlusion of the inner vestibule of the pore (Additional file 8: Figure S8d). On the other side, their symmetry-related counterparts in motif A are either oriented away from the pore region (Tyr66_A_ versus Tyr153_B_ and Gln59_A_ versus Met146_B_) or replaced by Ala residue (Ala49_A_ versus Arg137_B_) (Additional file 8: Figure S8e). The insertion of Pro57 (in a *cis* configuration) within the M2–M3 loop region directs the side chains of Gln59 and Tyr66 away from the pore region to create an open cavity on the extracellular side (Additional file 8: Figure S8e). Thus, these differences between motifs A and B result in the asymmetric status of the *Ss*TRIC channel with its outer vestibule open and inner vestibule closed.

On the cytoplasmic side, the region between the M5 and M6 helices forms a loop-helix-loop motif (residues Asn142-Tyr153) (Fig. 4b). It plugs in the inner vestibule of the hourglass-shaped pore and contributes to the formation of the first two bottlenecks (G1 and G2 gates shown in Fig. 3c) within the cytoplasmic vestibule. This plug-like motif binds to Asp97, Arg137, and Asp138 residues lining the surface of the pore lumen, through hydrogen bonds and ionic interactions (Fig. 4c). Thereby, it is stabilized in a position to block the access of ions into the pore (Fig. 4a). Removal of the plug-like motif from the structure generates a wide-open cavity in the cytoplasmic vestibule region (Fig. 4d). In this model, the open cytoplasmic vestibule becomes closer to the pore center and approaches the bottom of the extracellular vestibule.

**Fig. 5:**
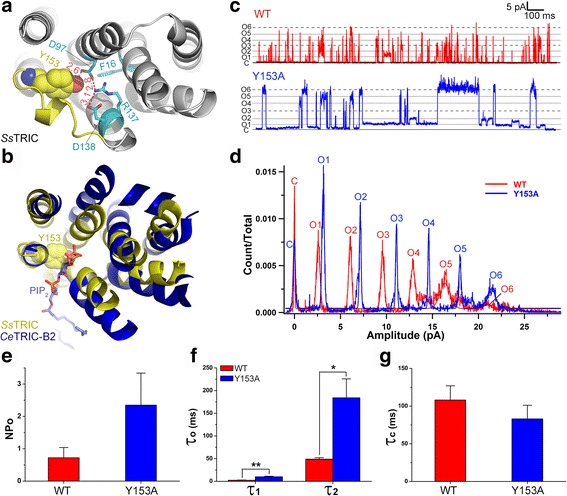
Effects of Y153A mutation on the electrophysiological behaviors of *Ss*TRIC channel. **a** Tyr153 and the surrounding residues in *Ss*TRIC. The plug motif is highlighted in *yellow*. Tyr153 is highlighted as a van der Waals (*VDW*) model, and the residues surrounding Tyr153 are shown as *cyan* stick models. The *red dashed lines* with distances (Å) labeled nearby indicate hydrogen bond or ionic interactions between two adjacent residues. The view is from the cytoplasmic side. **b** Superposition of a *Ss*TRIC monomer with a *Ce*TRIC-B2 monomer. The protein backbones are shown as cartoon models in *yellow* (*Ss*TRIC) or *blue* (*Ce*TRIC-B2). The PIP_2_ molecule in *Ce*TRIC-B2 is presented as a stick model in *blue* and *red*. PDB code: 5EIK for *Ce*TRIC-B2. **c** Representative electrophysiological recordings of the wild-type *Ss*TRIC (*red*) and Y153A mutant (*blue*) measured at +60 mV. **d** The all-points amplitude histogram of the Y153A (*blue*) and wild-type (*red*) data. The presence of six open-state peaks indicates these patches likely contain two trimers of Y153A/wild-type *Ss*TRIC channels. The bin width is set at 0.03 pA/bin. The histograms were both fitted with seven Gaussian functions. C, O1–O6 represent the closed state and opening of one to six monomers. **e** Open probability (*NP*_o_) analysis on the Y153A mutant (*blue*) and wild-type channel (*red*). *NP*_o_ = *t*_o_/*T*, where *t*_o_ is the total time that the channel is observed in the six open states and *T* is the total recording time. For the wild type, *n* = 5; for the mutant, *n* = 3. See Additional file 11 for the supporting data values. **f** and **g** Dwell time analyses on the open state (O1) (**f**) and closed state (**g**) of the Y153A mutant (*blue*) and wild-type channel (*red*). The * and ** symbols indicate ae *P* value <0.05 and 0.01 by *t* test, respectively. The standard errors of the mean values (*SEMs*) are indicated by the error bars

Among the residues from the plug motif, Tyr153 is closest to the pore center near Phe16, and forms a strong hydrogen bond with Asp97 (Fig. 5a). Strikingly, the position of the Tyr153 bulky side chain coincides with that of the inositol 4,5-biphosphate head group of the PIP_2_ molecule in *Ce*TRIC-B1, when the structure of *Ss*TRIC is superposed with that of *Ce*TRIC-B1 (Fig. 5b). In *Ce*TRIC-B1, the head group of the PIP_2_ molecule stabilizes the cytoplasmic gate through binding to two basic residues on the pore lumen [22]. Likewise, Tyr153 in *Ss*TRIC may have a similar role in controlling the cytoplasmic entrance through its bulky side chain, and may behave like a plug to the hourglass bottleneck. The electrophysiological analyses on the Y153A mutant of *Ss*TRIC confirm its role as a crucial gating element of the channel. The mutant appears more active than the wild type (Fig. 5c). Remarkably, the right shift of the amplitude histogram peaks of Y153A in respect to those of the wild type indicates that the conductance of the Y153A mutant is larger than that of the wild type (Fig. 5d). Moreover, the open probability (*NP*_*o*_) of Y153A is also higher than that of the wild type (Fig. 5e). The open state of the Y153A mutant also has a longer lifetime than that of wild type (Fig. 5f), while the closed-state lifetime remains very similar (Fig. 5g). This indicates that the Y153A mutation gives the channel an increased stability at the open state. Taken together, the electrophysiological behaviors of the Y153A mutant and the structural observations both demonstrate that Tyr153 has a pivotal role in the gating process of the *Ss*TRIC channel. It acts as a tethered plug being latched onto the surface of the pore lumen that occludes the pore from the cytoplasmic side.

## Discussion

As shown in Additional file 5: Figure S5d and e, the site for TRIC channels to select and bind monovalent cations (K^+^/Na^+^) during their permeation through the pore is most likely located at the central region around the two kinks on the M2 and M5 helices according to the following evidences. Firstly, two discrete water-/ion-binding sites (P0 and P0′) have been located in this region, and one of them (P0′) is accessible to a K^+^ surrogate (Tl^+^). Secondly, the coordination bond lengths between the ion on P0′ and the surrounding ligands are consistent with the K^+^-ligand bond length values observed in the KcsA or valinomycin structures (Additional file 5: Figure S5a–c). Thirdly, the kinks on M2 and M5 are general features conserved in the structures of both prokaryotic and eukaryotic TRIC channels. Such an asymmetric filter-like structure is evidently different from those of KcsA (or other homeo-tetrameric K^+^ channels) and may represent a new type of monovalent cation-binding site for ion channels. In this filter, K^+^ was bound in a monohydrated state and forms a trigonal geometry with its ligands, resembling half of the trigonal-antiprism K^+^ coordinations in valinomycin (Additional file 5: Figure S5a and b). For comparison, the filter in KcsA contains four consecutive layers of discrete K^+^-binding sites with a square antiprism geometry, and the K^+^ ion is bound in the center (Additional file 5: Figure S5f). Such a multilayer symmetrical filter in KcsA is more selective (for K^+^) than the single-layer asymmetric filter in the TRIC channel. The *P*_K_/*P*_Na_ permeability ratio for the KcsA channel is 166.7 or higher [32], whereas the *P*_K_/*P*_Na_ ratio of the *Ss*TRIC and mammalian TRIC channels is much lower at 1.21 and 1.5 [5], respectively. Lastly, the F104A mutant of *Ss*TRIC has a lower *P*_K_/*P*_Na_ ratio compared to the wild type, indicating that the mutant becomes less selective between K^+^ and Na^+^. The result suggests that the geometry of the fourth pore constriction site (G4) shaped by the bulky side chain of Phe104 and M2/M5 kinks is likely crucial for tuning the relative selectivity of *Ss*TRIC channel for K^+^ versus Na^+^.

The plug regions in prokaryotic TRIC orthologs contain a consensus “PX_5-7_E(D/Q)XYA” motif, while the regions interacting with the plug comprise two consensus motifs, namely the “**D**A(T/S)XGL” motif on M4 and the “GGXX**RD**” motif on M5 (the bold residues are those involved in binding the plug). Such conservation suggests that the Velcro-like plug-pore interactions might serve as a general gating mechanism for the prokaryotic TRIC orthologs. These three consensus motifs have become diversified in the eukaryotic members. The comparison between *Ss*TRIC and a *Ce*TRIC-B channel reveals an unexpected diverse feature of the pore plugs among the prokaryotic and eukaryotic members of the family (Additional file 9: Figure S9a and b). While the prokaryotic orthologs utilize a built-in plug in the M5–M6 loop region to control their cytoplasmic gates, the eukaryotic members have evolved a different strategy by adopting a lipid molecule, namely phosphatidylinositol 4,5-bisphosphate (PIP_2_), as the plug to their pores. The head group of PIP_2_ latches onto two basic residues on the pore lumen surface and occludes the pore from the cytoplasmic side (Additional file 9: Figure S9b). The M5–M6 loop region in the eukaryotic TRIC proteins turns into an irregular structure capping over the PIP_2_ head group, instead of forming a plug motif itself. In spite of these evident differences between prokaryotic and eukaryotic members of TRIC family, they share a common overall channel architecture and are probably unified by a general gating mechanism involving a Velcro-like plug-pore interaction. A model accounting for the potential gating mechanism of *Ss*TRIC has been summarized in Additional file 10: Figure S10.

The previous bioinformatics study suggested potential roles of prokaryotic TRIC orthologs in the efflux of metabolites [23]. This prediction awaits verification by further experimental evidences to demonstrate the physiological relevance of TRIC channel function in the transport processes of metabolites. A recent work on *Rs*TRIC and *Ss*TRIC indicated that they are involved in K^+^ uptake in *E. coli* cells [24]. Expression of *Ss*TRIC on the *E. coli* membrane leads to moderate inhibition of cell growth, resembling the phenotype of a gain-of-function ion channel (Additional file 6: Figure S6b). It is likely constitutively active on the *E. coli* membrane when a resting potential at −220 to −140 mV is present at different growth phases [33]. Putatively, the prokaryotic TRIC members might serve as membrane-potential regulators to prevent imbalance of resting potential (by providing K^+^ flux) and to maintain ion homeostasis in the cytoplasm of prokaryotic cells, instead of serving as a metabolite transporters themselves. Such a role may serve to regulate the active transport processes of metabolites across the membrane indirectly, as many transporters are driven by the electrochemical potential across the membrane [34].

## Conclusions

In summary, the high-resolution view of ion- and water-binding sites in the *Ss*TRIC channel unravels the presence of an asymmetric filter-like structure buried in the middle of an hourglass pore. The Velcro-like plug-pore interacting model derived in this study may offer a unified framework for understanding the gating mechanism of both prokaryotic and eukaryotic TRIC channels.

## Methods

### Cloning, protein expression, and purification

The gene encoding *Ss*TRIC was synthesized (GenScript) with optimized codon usage for protein expression in *Escherichia coli* and inserted between the *Nde*I and *Xho*I sites in the pET21b vector. The C41 (DE3) *E. coli* strain was used for protein expression. For large-scale expression, the overnight starter culture was inoculated into Terrific Broth (TB) media at a 1:40 (v:v) ratio. The cells were grown to optical density OD_600_ = 1.0 and then induced with 1 mM isopropyl β-d-1-thiogalactopyranoside (IPTG) at 37 °C for 2 h before being harvested through centrifugation.

For the purification of *Ss*TRIC protein, 10 g of defrosted cells were suspended in 100 ml of lysis buffer (50 mM Tris-HCl pH 8.0, 200 mM NaCl, 10 mM imidazole). After homogenization, 1.5 g of dodecyl-β-d-maltoside (DDM) was added to the suspension to extract the membrane proteins, and the mixture was stirred on ice for 30 min. The preparation was sonicated for 2 min (1 s on and 5 s off) in an ice-water bath. The cell lysate was centrifuged for 30 min at 16,000 RPM, 4 °C in a JA-25.50 rotor (Beckman). The supernatant was collected and applied by gravity to a column with 2 ml Ni-NTA superflow resin (Qiagen). After all the sample had flowed through, the column was washed with 10 ml of equilibration buffer (25 mM Tris-HCl pH 7.5, 200 mM NaCl, 20 mM imidazole, 0.5% DM) and then 10 ml of washing buffer (25 mM Tris-HCl pH 7.5, 200 mM NaCl, 80 mM imidazole, 0.4% DM). After the elution buffer (25 mM Tris-HCl pH 7.5, 200 mM NaCl, 300 mM imidazole, 0.4% DM) was applied, the fractions with protein concentration above 0.3 mg ml^–1^ were pooled and concentrated to 10–15 mg ml^–1^ in a 50 kDa cutoff Amicon Ultra-4 centrifugal filter unit (Millipore). Next, trypsin was added to the concentrated *Ss*TRIC protein sample at a 1:1000 (m:m) ratio and incubated at 37 °C for 1 h for limited proteolysis treatment. The product was then applied to a Superdex 200 10/300 GL column (GE Healthcare) and eluted in a buffer containing 10 mM Tris HCl pH 7.5, 150 mM NaCl, and 1.1% OG. The major peak fraction of the eluted protein was pooled and concentrated to ~10 mg ml^–1^ for the crystallization experiments.

### Crystallization, crystal soaking, data collection, and processing

The initial crystallization condition was identified through sparse matrix screening using the MemGold kit (Molecular Dimensions). The optimized recipe for crystallization of *Ss*TRIC in OG involves setting up hanging drops with 1 μl 10–15 mg ml^–1^ protein sample, 1 μl well solution (22–26% PEG 3000, 0.1 M Tris-HCl pH 8.0, 0.2 M sodium acetate, and 0.2 M KCl), and 0.2 μl 30% ethylene glycol. The drops were equilibrated against 0.5 ml of well solution at 16 °C. The best crystals were small hexagonal plates (0.1–0.3 mm in diagonal length) grown on the surface of the drops. To prepare crystal samples with only K^+^, the plate crystals initially grown in the mixture of NaAc and KCl were washed extensively in a stabilizing solution with KCl as the only salt (26% PEG 3000, 50 mM Tris-HCl pH 8.0, 1.1% OG, 0.1% DM, 3% ethylene glycol, 0.5 M KCl). These crystals were further soaked in the same solution at 16 °C for ~24 h before being harvested. The Tl^+^-containing crystals were prepared by washing in a stabilizing solution with 0.5 M KNO_3_ to remove Cl^–^ ions. Otherwise, they would have formed a precipitate with Tl^+^ ions because TlCl has low solubility in water. Next, the crystals were washed and soaked in a new stabilizing solution with 0.5 M TlNO_3_ for 24 h.

For phasing, the crystals of A15C or A44C mutants were grown under the same conditions as for the wild type and derivatized with 1 mM CH_3_HgCl for 24–48 h. The mutation was introduced through the QuikChange Site-Directed Mutagenesis method (Stratagene). The high-resolution KCl dataset (Table 2) was collected at the BL17U of the Shanghai Synchrotron Radiation Facility (SSRF) equipped with an ADSC Q315 CCD detector. The anomalous diffraction data of the Tl^+^-containing crystals were collected at the home source with a Rigaku Micromax-007 rotating anode generator and a Rigaku IP IV++ detector. For data processing, the iMOSFLM [35] or HKL2000 [36] program was used.

**Table 2:** Data collection, phasing, and structure refinement statistics of *Ss*TRIC

Data collection	CH_3_HgCl (A15C mutant)	KCl (wild type)	TlNO_3_ (anomalous)
Wavelength (Å)	1.00000	1.00137	1.5418
Beamline^a^	SSRF BL17U	SSRF BL17U	Rigaku MicroMax-007
Space group	*C* 2	*C* 2	*C* 2
Resolution (Å)	50–2.5 (2.64–2.5)	50–2.2 (2.32–2.2)	50–3.4 (3.52–3.4)
Cell dimensions (Å, °)	*a* = 152.07	*a* = 152.07	*a* = 150.40
	*b* = 87.48	*b* = 87.73	*b* = 86.44
	*c* = 173.62	*c* = 173.06	*c* = 173.30
	*β* = 108.75	*β* = 108.90	*β* = 108.59
*R*_*sym*_	0.211 (0.883)	0.149 (0.604)	0.133 (0.886)
*R*_*pim*_^b^	0.076 (0.308)	0.075 (0.300)	N/A
*I/σ*	8.1 (3.9)	7.1 (2.7)	12.1 (2.1)
Completeness (%)	98.8 (96.9)	97.6 (94.8)	88.6 (87.2)
Redundancy	8.9 (8.7)	4.7 (4.6)	5.9 (6.0)
Phasing statistics			
No. of heavy atom sites	6		
Figure of merit (before/after DM)	0.258 (0.640)		
Refinement statistics			
Resolution		50–2.2	
No. of reflections (no. of reflections in free set)		106,561 (2614)	
*R*_*work*_ (%)		21.08	
*R*_*free*_ (%)		22.85	
No. atoms (B-factors, Å^2^)			
All atoms		9642 (38.1)	
Protein		9054 (37.0) (6 chains)	
Cation		33 K^+^ (77.9)	
Water		381 (46.0)	
Others (detergent)		174 (68.4)	
Ramachandran plot (%)^c^		96.8/2.5/0.7	
RMSD bond length (Å)		0.007	
RMSD bond angles (°)		1.22	

The *R*_*sym*_, *R*_*pim*_, *I/σ*, completeness, and redundancy are presented as the statistics for overall and outmost shell (in parentheses)

^a^ *SSRF* Shanghai Synchrotron Radiation Facility

^b^ *R*_*pim*_ is the precision-indicating (multiplicity-weighted) *R*_*sym*_ reported by the Scala program. *R*_*pim*_ = ∑_*hkl*_[1/(*N* − 1)]^1/2^∑_*i*_|*I*_*i*_(*hkl*) − 〈*I*(*hkl*)〉|/∑_*hkl*_∑_*i*_ *I*_*i*_(*hkl*) The HKL2000 program used to process TlNO_3_ data did not report *R*_pim_ statistics

^c^Percentage of residues in most favored, additional allowed, and generously allowed regions in Ramachandran plot. No residues are observed in the disallowed region

### Structure determination, refinement, and analysis

The structure was solved by the single-wavelength anomalous dispersion method using the Autosol program in the PHENIX suite [37]. The anomalous diffraction dataset of Hg-labeled A15C crystal collected at 1.00000 Å wavelength was used for phasing. Six Hg atoms were located in one asymmetric unit, and the initial set of selected area diffraction (SAD) phases has a figure of merit (FOM) of 0.258 which is further improved to 0.640 through density modification. The initial model automatically generated by the Autobuild program in PHENIX contains a nearly complete polypeptide structural model with *R*_work_ = 0.250 and *R*_*free*_ = 0.260. This model was further improved by iterative cycles of manual adjustment in Coot [38] and refinement in CNS 1.2 [39] using the high-resolution (2.2 Å) KCl-only data. The longest polypeptide chains were continuously traced from Met 1 to Pro 198. From the electron density map, it appears that the limited proteolysis by trypsin removed only seven residues at the carboxy-terminal region after Pro198 and the hexa-histidine tag region. The transmembrane domain and the loop region remain intact after proteolysis. The K^+^ ions were modeled and cross-validated by the anomalous difference Fourier peaks of the Tl^+^ ion. The anomalous difference Fourier map of Tl^+^-containing crystals was computed by the FFT program in the CCP4 suite [40]. The data collection, phasing, and structure statistics are summarized in Table 2.

For the structural analysis, the PROMOTIF program [41] was used to analyze the secondary structures, Lsqman [42] was used to superpose different structures, HOLE [43] was used to probe the pores within the channel, PISA [44] was used to analyze protein interfaces and buried surfaces within the trimer, and the APBS [45] tool was used to calculate surface electrostatic potential. For sequence alignment, the output from the ClustalW [46] program was manually checked and readjusted. BOXSHADE 3.21 and ESPript programs [47] were used to generate sequence alignment figures. The cartoon structural figures were produced in PyMOL [48] or Chimera [49], and the electron density maps were displayed using Coot [38].

### Lipid extraction and identification

To extract lipids, purified *Ss*TRIC or *E. coli* TRIC (*Ec*TRIC) protein samples (200 μl 10 mg ml^–1^ protein in β-DM) were mixed with 180 μl of solvent with chloroform, methanol, and concentrated HCl solution (1:2:0.02, v/v/v). Subsequently, 60 μl of chloroform and 60 μl of 2 M KCl (sigma) were added to each tube. The mixture was vortexed and then centrifuged for 5 min at 2400 g to separate the organic phase from the aqueous phase. The organic phase was then separated through thin layer chromatography (TLC). At first, 20 ul organic phases extracted from *Ss*TRIC and *Ec*TRIC protein samples were spotted on the TLC silica gel plate (Merck). As reference standards, triacylglycerol (TG, C18:1), diacylglycerol (DG, C16:0), monoacylglycerol (MG, C16:1), palmitic acid, and steric acid samples were applied on the same plate. The solvent used for TLC contains *n*-hexane, diethyl ether, and acetic acid (70:30:1, v:v:v). After being separated on the TLC plate, the lipid fractions were visualized by spraying the plate with 0.5% iodine-chloroform. To further identify the lipid fraction, mass spectroscopy was performed under the electrospray ionization (ESI) positive and negative scan modes. As a control, the organic phase extraction of the blank elution buffer used for protein purification was also examined through mass spectroscopy under the same modes.

### Reconstitution of *Ss*TRIC in small unilamellar vesicles (SUVs)

A lipid mixture contraining 90% 1,2-diphytanoyl-*sn*-glycero-3-phosphocholine (DPhPC) and 10% cholesterol (w/w) in chloroform was dried under vacuum in a CentriVap Concentrator (Labconco) for 4 h. The lipid sample was then suspended at 10 mg ml^-1^ in a low-salt buffer (1 mM 4-(2-hydroxyethyl)-1-piperazineethanesulfonic acid (HEPES) pH 7.2, 5 mM KCl). SUVs were formed by tip sonication (50 Hz, 1 s on, 1 s off for 1 min) and then presolubilized by 10 mM DM for 30 min at RT. Subsequently, the *Ss*TRIC protein sample was added to the presolubilized SUVs to achieve a protein:lipid ratio of 1:10 (m:m). More DM was added to a final concentration of 17.5 mM, and the resulting mixture was gently agitated for 1 h at room temperature. Detergent was removed by dialysis in the low-salt buffer (1 mM HEPES pH 7.2, 5 mM KCl). The external buffer was changed every 12 h for 3 days. After dialysis, the resulting SUVs were aliquoted, flash-frozen in liquid nitrogen, and stored at −80 °C.

### Preparation of giant unilamellar vesicles (GUVs) for electrophysiology

The GUV samples were generated by the electroformation technique using the Nanion Vesicle Prep Pro device (Nanion). Before electroformation, trehalose was added to the preformed SUV solution to a final concentration of 10 mM in order to protect the *Ss*TRIC protein during the partial dehydration process. About 10 μl of SUV solution was applied in small droplets (~0.2 μl/droplet) on the indium tin oxide (ITO)-treated glass slide. The droplets were left to dry in the open ﻿air﻿ for approximately 30 min at room temperature. Subsequently, 300 μl of 1 M sorbitol solution was carefully added onto the lipid film, and a cassette sandwiching the sample in the middle was assembled. During assembly, we ensured that the ITO layers of the slides were facing and that they touched the sample. For the electroformation process, the protocol was set as 0.1 to 1.0 V at 12 Hz frequency for 3 h. For the next step, the frequency was lowered to 4 Hz and the voltage was raised to 2 V for 30 min to detach the GUVs from the glass slides. The temperature was kept constant at 36 °C throughout the electroformation process. Alternatively, the purified protein can be reconstituted directly on the GUVs (protein:lipid = 1:200–250, w:w; lipid: 95% azolectin + 5% cholesterol, w:w) prepared through a modified sucrose method [50]. In some cases when the GUVs did not attach well to the bottom of the sample chamber, the lipid composition was adjusted by adding DPhPC to the mixture at a cholesterol:DPhPC:azolectin (w:w:w) ratio of 1:5:17 or 1:10:15.

### Electrophysiology

All recordings were performed with the inside-out configuration. The intracellular side of the GUV membrane was exposed to the bath solution, and the extracellular side was exposed to the pipette solution. The bath and pipette solutions contained 210 mM KCl and 10 mM HEPES (pH 7.2). Patch pipettes with resistances of 8–9 MΩ were used, and the patch resistance increased to ~2 GΩ after the pipette sealed tightly with the GUV membrane. For recording the data under bi-ionic conditions, the pipette solution was kept constant with 210 mM KCl and 10 mM HEPES (pH 7.2), while the bath solutions were: 210 mM NaCl and 10 mM HEPES (pH 7.2) for *P*_K_/*P*_Na_ analysis; 210 mM KCl, 75 mM CaCl_2_ or MgCl_2_, and 10 mM HEPES (pH 7.2) for the test of Ca^2+^/Mg^2+^ and Cl^-^ permeability. Single-channel recordings were made with an EPC-10 amplifier (HEKA, Lambrecht, Germany) under different voltage settings at 50 kHz with a 0.5-kHz filter and a 50-Hz notch filter. All experiments were done at room temperature (21–24 °C).

The Clampfit Version 9.0 (Axon Instruments, Foster City, CA) was used for data analysis, Excel Version 2010 (Microsoft) and OriginPro 8 were used for statistical analysis, and Igor Pro 6.32A (WaveMetrics, USA) was used for graphics. The single-channel conductance was obtained through linear fitting of the current-voltage plots. The statistical data are reported as mean value ± SEM. The Student’s *t* test was used to assess statistical significance; *n* represented the number of experiments analyzed.

### Cell-based functional assay

The plasmids carrying *Ss*TRIC mutants were transformed into the C41 (DE3) *E. coli* strain for protein expression and in vivo functional assay. For the measurement of cell growth curves, single colonies of the *E. coli* transformants were used to inoculate 3 ml of liquid LB media plus 100 μg/ml ampicillin and grown at 37 °C for 2–3 h. The cell densities of the cultures were normalized to OD_600_ = 0.1 with LB media. Subsequently, 75 μl of diluted cell culture was added into the wells of a 96-well plate and mixed with 75 μl LB media with 1 mM IPTG added. The plate was then sealed with the CyclerSeal film (Platemax), and the growth of cells at 37 °C within each well was monitored continuously in the Thermo Varioskan Flash Plate Reader. The OD_600_ was taken every 20 min while the plate was shaken at 300 cycles of horizontal shakes per minute (SPM).

The protein expression was analyzed through western blots on the membrane fractions of the *E. coli* cells harvested after being induced by IPTG. To prepare the membrane fractions, 1 g of defrosted cells was suspended in 5 ml lysis buffer (50 mM Tris-HCl pH 8.0, 200 mM NaCl, 10 mM imidazole). Lysozyme was added to a final concentration of 1 mg ml^-1^, and the suspension was stirred at 4 °C for 1 h. The preparation was sonicated for 2 min in an ice-water bath with a program setting of 1 s on and 5 s off. The cell lysate was spun at 14,800 RPM, 4 °C for 30 min. The supernatant was collected and centrifuged at 14,800 RPM, 4 °C for another 30 min to remove large insoluble debris. Subsequently, the supernatant (1 ml) was ultracentrifuged at 38,000 RPM (~100,000 × g) in an S140AT rotor (Hitachi) at 4 °C for 2 h. The pellet was resuspended in 1 ml of washing buffer (50 mM Tris-HCl pH 8.0, 200 mM NaCl, 10 mM imidazole) and ultracentrifuged again. The membrane pellets were collected and weighted, followed by resuspension in the washing buffer to a final concentration of 50 mg ml^-1^. The samples were mixed with 5 × SDS loading buffer and then separated by electrophoresis on 12% SDS-PAGE gel. For western blot analysis, the protein bands on the gel were transferred to a nitrocellulose membrane. After blocking and washing, the blot was probed by HRP-conjugated anti-Histag mAb antibody (GenScript, cat. number: A00612, lot number: 16B001004, RRID: AB_915573) and then developed with the Western Lightning ULTRA substrate (PerkinElmer).

## Additional files

Additional file 1: Figure S1. Superposition of *Ss*TRIC structure with two previous structures of prokaryotic TRIC orthologs. **a** and **b** *Ss*TRIC trimer and monomer superposed on the structure of *Ss*TRIC-Fab complex (Protein Data Bank (PDB):5H35). The Fab antibody fragment is omitted for clarity. **c** and **d** *Ss*TRIC trimer and monomer superposed on the structure of *Rs*TRIC (PDB:5H36). Protein backbones are represented as ribbon cartoon models. *Color code: silver*, *Ss*TRIC structure reported in this work; *blue*, *Ss*TRIC in complex with Fab; *magenta*, *Rs*TRIC structure. PDB codes: *Ss*TRIC-Fab complex, 5H35; *Rs*TRIC, 5H36. Triacylglycerol is shown as *yellow* ball-and-stick model, while the acyl chains of lipid molecules in *Ss*TRIC-Fab and *Rs*TRIC are presented as *green* ball-and-stick models. (TIF 1762 kb)

Additional file 2: Figure S2. Comparing the thickness of transmembrane domains of *Ss*TRIC, *Ce*TRIC-B, and RyR1. PDB codes: *Ce*TRIC-B1, 5EGI; RyR1, 3J8H. The surface models (carbon in *silver*, oxygen in *red*, and nitrogen in *blue*) are shown on the *top layer*, while cartoon models are shown on the *lower layer*. The two *horizontal lines* indicate the estimated location of membrane surfaces. (TIF 1627 kb)

Additional file 3: Figure S3. The pore structures of *Ss*TRIC compared to those of *Rs*TRIC and *Ce*TRIC-B1. **a** *Ss*TRIC, **b** *Rs*TRIC, **c** *Ce*TRIC-B1. PDB codes: *Rs*TRIC, 5H36; *Ce*TRIC-B1, 5EGI. Color codes for the pore profiles based on the data output by HOLE program: *red*, low radius surface (<1.5 Å); *green* areas, normal pore surface (1.5–2.3 Å); *light blue* areas, high radius surface (>2.3 Å). The protein backbones are shown as ribbon models. The key amino acid residues shaping the constriction areas along the pore are highlighted as *green* stick models, while the PIP_2_ in *Ce*TRIC-B1 is shown as *yellow* and *red* sticks. (TIF 891 kb)

Additional file 4: Figure S4. Electron density maps of the pore region in *Ss*TRIC channel. **a** 2*F*_o_-*F*_c_ electron densities of the pore region contoured at 1.5 × σ level. The refined structures are shown as stick models superposed on the map. The water molecules and K^+^ ions are displayed as *red* and *silver bullets*, respectively. **b** The same map zoomed in around the pore center to show the local view of the P0 and P0′ sites encircled by the M2 kink, M5 kink, Phe16, and Phe104. **c** The anomalous difference Fourier peaks of the Tl^+^ ions bound to the *Ss*TRIC channel. The map at 3.4-Å resolution is contoured at 3 × σ level and shown as *magenta meshes* superposed on the C_α_-trace model of a *Ss*TRIC trimer. The images shown are stereo pairs, and the view is approximately perpendicular to the C3 axis. The Tl-1 and Tl-2 peaks are located at the sites corresponding to P0′ and P3′ sites shown in **a**. The relatively weaker densities of Tl-1 and Tl-2 peaks compared to those on surfaces indicate that the occupancy of Tl^+^ ions on these internal sites is much lower than on the surface ones. Note that only one monomer in the trimer contains a peak above 3 × σ level at P0′ site (Tl-1), suggesting that this site at the current conformational state is not yet fully accessible to Tl^+^/K^+^. Stereo pairs are shown in **a**–**c**. (TIF 7223 kb)

Additional file 5: Figure S5. Central putative monovalent cation-binding site and filter-like structure in *Ss*TRIC compared to those of valinomycin and KcsA channel. **a** The ligands around the putative K^+^ (*purple sphere*, likely with partial occupancy and mixed with water molecules) at pore center of *Ss*TRIC. Zoom-in view shows the trigonal coordination mode of K^+^; the side chain of Phe104 is located below K^+^. *Dashed lines* indicate the close interactions between K^+^ and its ligands with the bond length (Å) labeled nearby. The interaction between K^+^ and the backbone amide is not uncommon, as some similar interactions are found in the structure of gramicidin (PDB code 2IZQ, the K^+^-NH bond length is 3.1–3.4 Å, slightly weaker than the one we observed.). **b** The trigonal antiprism-type coordination of K^+^ in valinomycin (Cambridge Crystallographic Data Centre/CCDC accession code: VALINK). DVA and V indicate d- and l-valines, respectively. *Gray dashed lines* are the coordination bonds absent in *Ss*TRIC. **c** The square antiprism-type coordination of K^+^ in KcsA. (PDB:1K4C). In panels **a**–**c**, K^+^ ions and water molecules are shown as *purple* and *red spheres*, respectively. The amino acid residues coordinating K^+^ ions are highlighted as ball-and-stick models. **d** and **e** The single layer filter-like structure sandwiched among M2 kink, M5 kink, Phe104, and Phe16 in *Ss*TRIC. The putative K^+^ ion and a nearby water molecule are shown as sphere models. Amino acid residues (Phe16, Thr41, Gly45, Phe104, Val129, and Gly133) involved in binding K^+^ (or Na^+^) and water binding are shown as stick models. View in **e** is rotated ~90° with respect to view in **a**. **f** The canonical four-layer filter structure in KcsA channel. *Purple funnels* in **e** and **f** indicate the presumed permeation pathways for monovalent cations. (TIF 1214 kb)

Additional file 6: Figure S6. In vivo assay on the function of *Ss*TRIC and various mutants. **a** Side view of the *Ss*TRIC monomer with the residues chosen for alanine-scanning mutagenesis highlighted in *cyan* or *yellow*. **b** Growth curves of *E. coli* cells expressing wild-type (*WT*) and various mutants of *Ss*TRIC. **c** Control data for the cell growth assays. *Ec*MscL: the large-conductance mechanosensitive channel (MscL) from *E. coli.* Overexpression of wild-type *Ec*MscL does not inhibit the cell growth, as it remains tightly closed at the resting state, while its gain-of-function mutant G26H has a dramatic inhibitory effect on cell growth. They are included as controls for comparison with the phenotypes of *Ss*TRIC and its mutants. Vector: empty pET21b vector used for expressing *Ss*TRIC and mutants, included as a negative control; the R187A mutation site is relatively distant from the gating region and thus serves as an internal control. The error bars represent the standard errors of mean values (SEM, *n* = 3). **d** Western blots of the membrane fractions of *E. coli* cells expressing wild-type and mutant *Ss*TRIC proteins. *M* molecular weight marker, *WT* wild-type *Ss*TRIC. The protein was probed by anti-Histag antibody (see Methods for details). For each lane, 80 μg wet membrane solubilized by SDS-PAGE loading buffer was loaded. (TIF 632 kb)

Additional file 7: Figure S7. Lipid molecules bound in the *Ss*TRIC homotrimer. **a** Thin layer chromatography of the lipid samples extracted from purified *Ss*TRIC and *Ec*TRIC preparations. Three major neutral lipid bands from purified *Ss*TRIC and *Ec*TRIC samples are visible on the plate upon being stained by iodine. **b** Mass spectrometry analysis of lipid samples extracted from purified *Ss*TRIC protein under electrospray ionization positive mode. Species with m/z at 987.70 may correspond to an ionized triacylglycerol molecule. **c** Mass spectrometry of the blank solution used for protein purification as a control. The species with m/z at 505.40 and 434.40 likely arise from the detergent. **d** Sectional view of *Ss*TRIC trimer along the C3 axis from cytoplasmic side. *Green meshes* are 2*F*_o_-*F*_c_ electron densities (contoured at +1.0 × σ level) potentially belonging to lipid cofactors. **e** Zoom-in view of a fatty acyl chain of TG molecule in *Ss*TRIC. The amino acid residues involved in binding TG are shown as stick models. **f** Sequence alignment of the M2 and M5 regions in various prokaryotic TRIC members. *Dark triangles* indicate the amino acid residues involved in binding lipid molecules. Ss *Sulfolobus solfataricus*, Rs *Rhodobacter sphaeroides*, Gf *Gramella forsetii*, Sa *Sulfolobus acidocaldarius*, Ms *Metallosphaera sedula*, Pn *Pyrobaculum neutrophilum*, Pi *Pyrobaculum islandicum*, Ec *Escherichia coli*, Hi *Haemophilus influenza*, Dg *Deinococcus geothermalis*, Tt *Thermus thermophilus*, Ta *Thermus aquaticus*. **g** Sectional view of *Ce*TRIC-B2 trimer (PDB:5EIK) along the C3 axis from cytoplasmic side. *Green meshes* are 2*F*_o_-*F*_c_ electron densities of PIP_2_ molecules (contoured at +1.0 × σ level). **h** Zoom-in view of the region around PIP_2_ in *Ce*TRIC-B2. **i** Sequence alignment of representative eukaryotic TRIC members in M2 and M5 regions involved in binding lipid acyl chains. Ce *Caenorhabditis elegans*, Hs *Homo sapiens*, Mm *Mus musculus*, Gg *Gallus gallus*, Dr *Danio rerio*, Xt *Xenopus tropicalis*. (TIF 2564 kb)

Additional file 8: Figure S8. Asymmetric features of the internal repeats in *Ss*TRIC monomer. **a** Structure of an *Ss*TRIC monomer represented as a cartoon ribbon model. Convex quadrilaterals in *light blue* and *pink* highlight the regions of motifs A and B, respectively. The *dark solid ellipse at the center* shows the position of a pseudo-C2 axis relating the two motifs. The view is along the pseudo-C2 axis running nearly parallel to the membrane plane. **b** Superposition of motif B (*golden*) with motif A (*blue*). **c** Alignment of the amino acid sequences of motifs A and B. Identical residues are in *dark boxes*; similar ones are in *gray backgrounds*. The *blue and golden arrows* highlight the key residues around the pore region. **d** The intracellular vestibule in motif B is occluded. **e** The extracellular vestibule of the pore harbored in motif A is open. The views are approximately along the pore axis. The key amino acid residues surrounding the pore region are shown as stick models. The *asterisk symbols* indicate the approximate location of the pore. (TIF 3075 kb)

Additional file 9: Figure S9. Comparison between *Ss*TRIC and *Ce*TRIC-B2 structures. **a** Structures of *Ss*TRIC and *Ce*TRIC-B2 monomers superposed and viewed from the same angle. The *Ss*TRIC (*left*) and *Ce*TRIC-B2 (*right*, PDB code: 5EIK) backbones are represented as cartoons, and the lipid cofactor (PIP_2_) and key amino acid residues are shown as sticks. The plug motif/molecule is highlighted in *cyan*, while those involved in binding the plug are colored in *yellow*. **b** The position of plug motif in *Ss*TRIC (*left*) compared to that of PIP_2_ in *Ce*TRIC-B2 (*right*). The key residues involved in binding the plug motif/PIP_2_ are represented as *yellow sticks*. (TIF 2656 kb)

Additional file 10: Figure S10. Proposed model accounting for the gating mechanism of *Ss*TRIC channel. At the closed state, the plug motif forms tight interactions with the pore lumen surface and blocks the cytoplasmic entrance. Upon activation by voltage, movement of the charged residues under electric field leads to destabilization of the interactions between plug motif and the potential voltage-sensing residues. The plug is thereby dislocated from the blocking position so that the cytoplasmic entrance of the hourglass-shaped pore is open for K^+^ ions to permeate through the pore. *Blue* and *red sticks* represent the positively and negatively charged residues, respectively; *purple spheres* represent K^+^ ions. For clarity, M3 and M7 helices are omitted in the model. For K_v_ or Na_v_ channels, they generally adopt a string of positively charged amino acid residues (Arg and Lys) on a transmembrane helix (S4 helix) of the voltage-sensing domain (*VSD*) to sense electrical signals [51–53]. While *Ss*TRIC channel does not contain a canonical VSD in its structure, it does have nine positively charged residues (Arg and Lys) distributed asymmetrically on two solvent-exposed surfaces (Fig. 4b), reflecting the positive-inside rule for membrane proteins [54]. The eight positively charged residues on the luminal side are not randomly distributed. Among them, six cluster around the cytoplasmic gate area, and three of them (Lys 27, Lys 23 on M1 helix and Arg137 on M5 helix) form an array of positively charged regions lining the interfacial groove between M1 and M5 (Fig. 4b). This array of positively charged residues is stabilized by three acidic residues: Asp97, Asp138, and Glu143 (Fig. 4c). Furthermore, three key residues from the plug motif interact directly or indirectly with these potential voltage-sensing residues (Fig. 4c). Hence, the Velcro-like structure may serve as a basis for the potential voltage-dependent regulation of *Ss*TRIC channel activity. (TIF 743 kb)

Additional file 11: Supporting data for Fig. 2 and 5e–g. (XLSX 19 kb)
