# Supplementary figures and images for: Ion- and water-binding sites inside an occluded hourglass pore of a trimeric intracellular cation (TRIC) channel
Source: BMC Biol. 2017 Apr 22;15:31. doi: 10.1186/s12915-017-0372-8 (PMC5401562; doi:10.1186/s12915-017-0372-8)

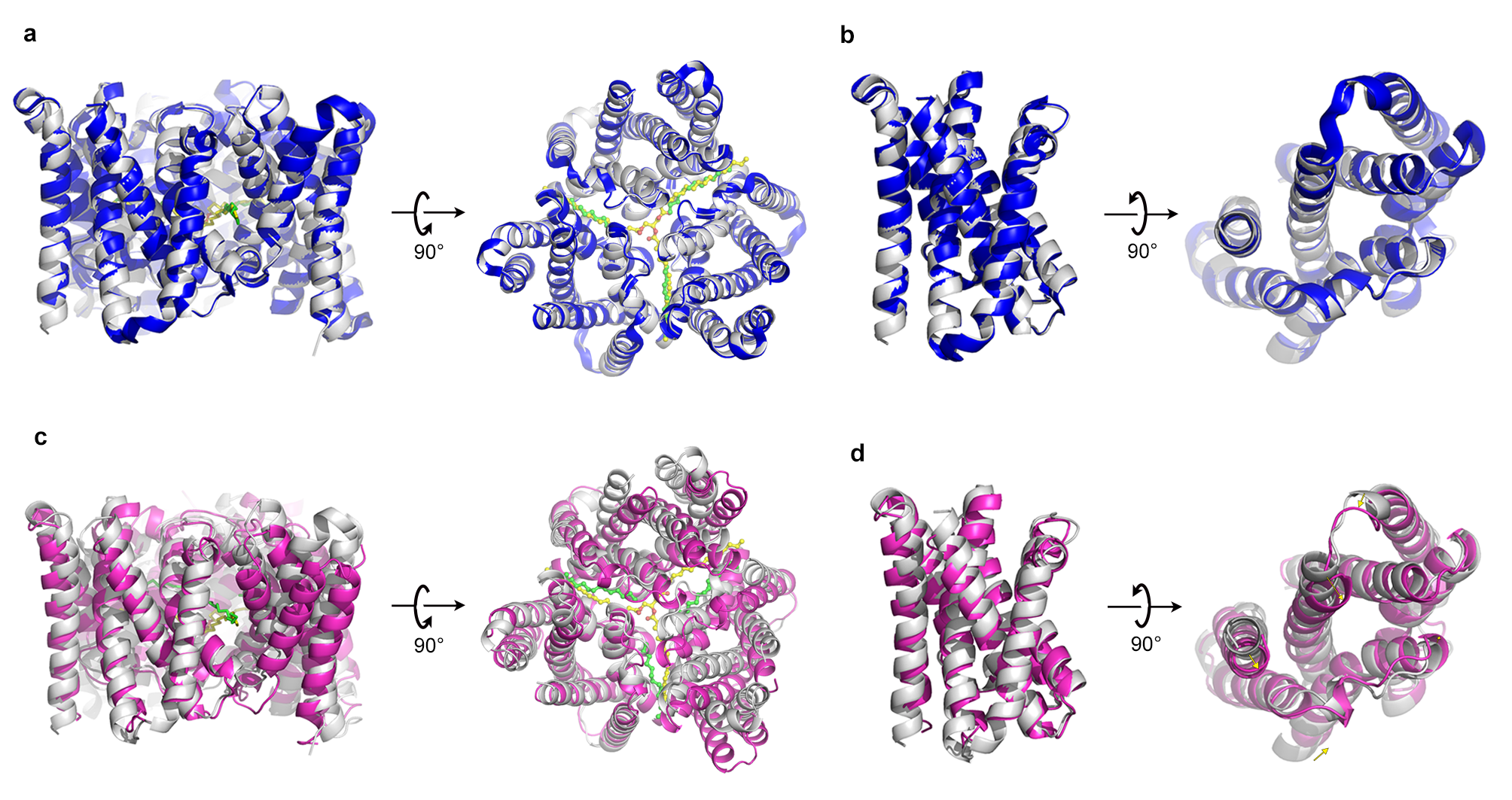

Supplement: Supplementary file 1 — Superposition of SsTRIC structure with two previous structures of prokaryotic TRIC orthologs. a and b SsTRIC trimer and monomer superposed on the structure of SsTRIC-Fab complex (Protein Data Bank (PDB):5H35). The Fab antibody fragment is omitted for clarity. c and d SsTRIC trimer and monomer superposed on the structure of RsTRIC (PDB:5H36). Protein backbones are represented as ribbon cartoon models. Color code: silver, SsTRIC structure reported in this work; blue, SsTRIC in complex with Fab; magenta, RsTRIC structure. PDB codes: SsTRIC-Fab complex, 5H35; RsTRIC, 5H36. Triacylglycerol is shown as yellow ball-and-stick model, while the acyl chains of lipid molecules in SsTRIC-Fab and RsTRIC are presented as green ball-and-stick models. (TIF 1762 kb) [file 12915_2017_372_MOESM1_ESM.tif]

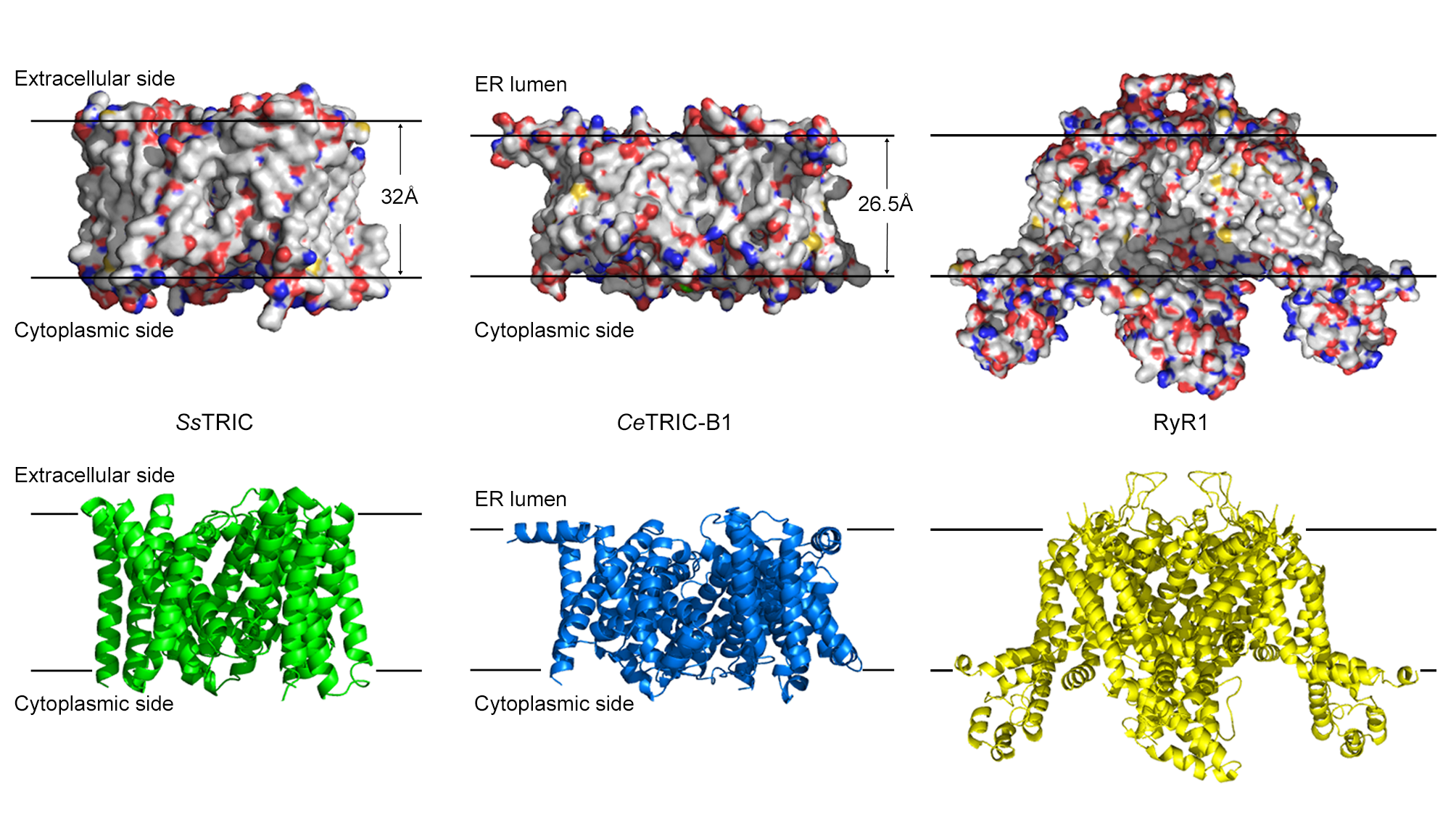

Supplement: Supplementary file 2 — Comparing the thickness of transmembrane domains of SsTRIC, CeTRIC-B, and RyR1. PDB codes: CeTRIC-B1, 5EGI; RyR1, 3J8H. The surface models (carbon in silver, oxygen in red, and nitrogen in blue) are shown on the top layer, while cartoon models are shown on the lower layer. The two horizontal lines indicate the estimated location of membrane surfaces. (TIF 1627 kb) [file 12915_2017_372_MOESM2_ESM.tif]

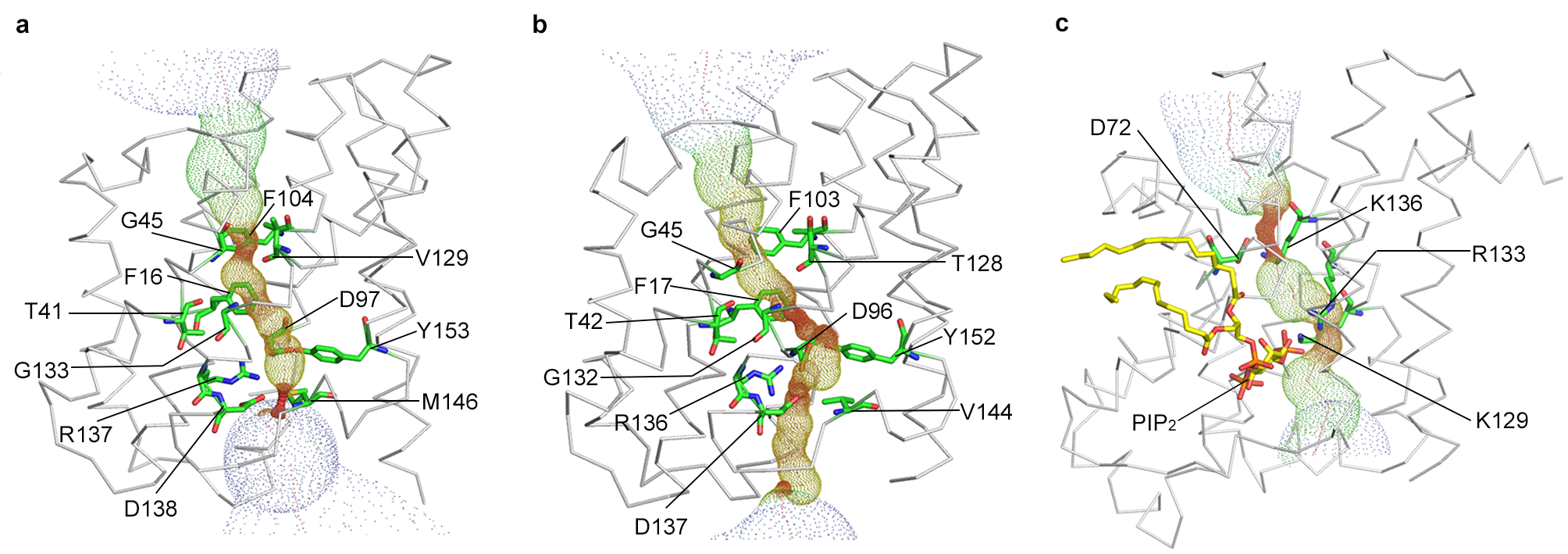

Supplement: Supplementary file 3 — The pore structures of SsTRIC compared to those of RsTRIC and CeTRIC-B1. a SsTRIC, b RsTRIC, c CeTRIC-B1. PDB codes: RsTRIC, 5H36; CeTRIC-B1, 5EGI. Color codes for the pore profiles based on the data output by HOLE program: red, low radius surface (<1.5 Å); green areas, normal pore surface (1.5–2.3 Å); light blue areas, high radius surface (>2.3 Å). The protein backbones are shown as ribbon models. The key amino acid residues shaping the constriction areas along the pore are highlighted as green stick models, while the PIP2 in CeTRIC-B1 is shown as yellow and red sticks. (TIF 891 kb) [file 12915_2017_372_MOESM3_ESM.tif]

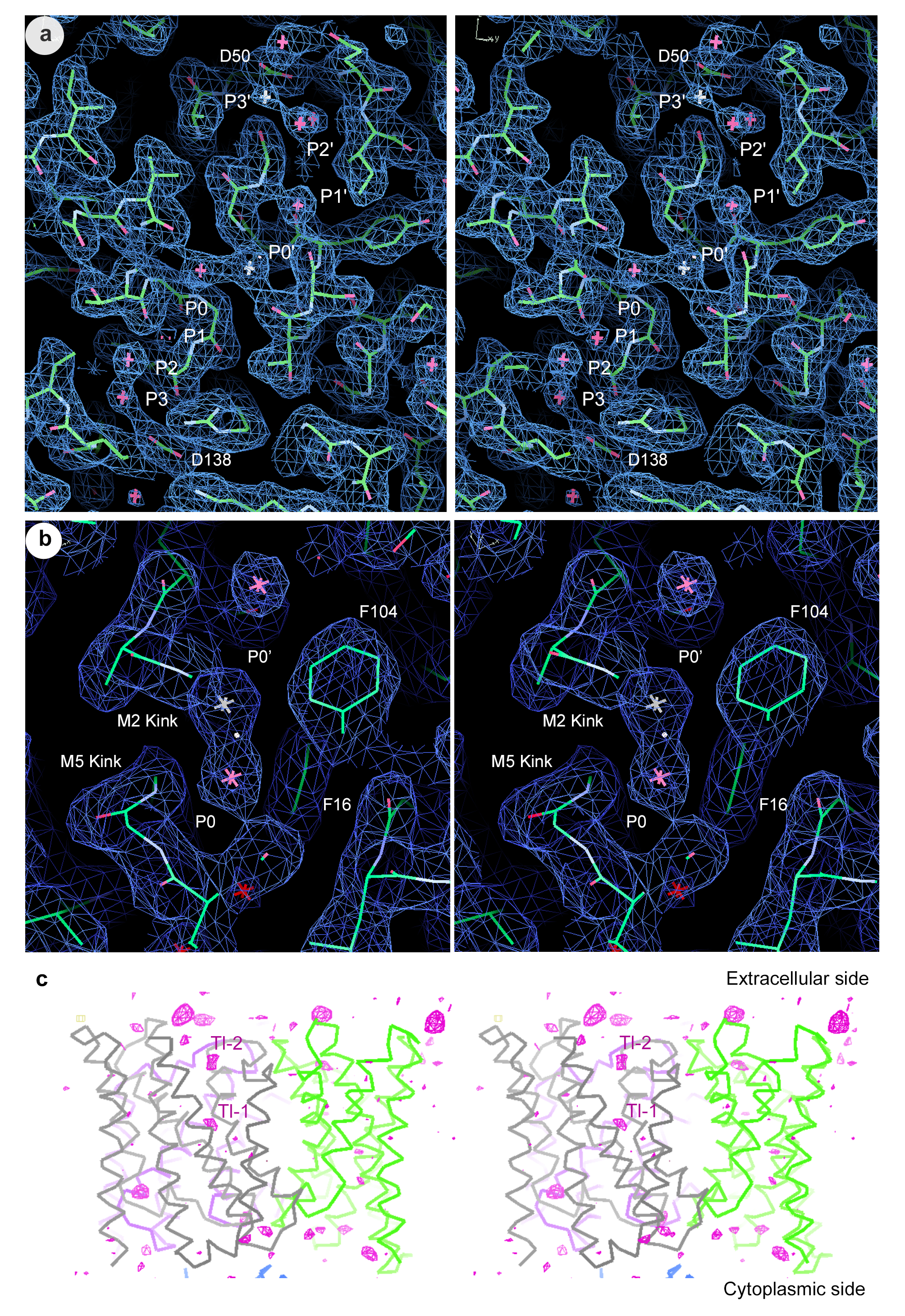

Supplement: Supplementary file 4 — Electron density maps of the pore region in SsTRIC channel. a 2F o-F c electron densities of the pore region contoured at 1.5 × σ level. The refined structures are shown as stick models superposed on the map. The water molecules and K+ ions are displayed as red and silver bullets, respectively. b The same map zoomed in around the pore center to show the local view of the P0 and P0′ sites encircled by the M2 kink, M5 kink, Phe16, and Phe104. c The anomalous difference Fourier peaks of the Tl+ ions bound to the SsTRIC channel. The map at 3.4-Å resolution is contoured at 3 × σ level and shown as magenta meshes superposed on the Cα-trace model of a SsTRIC trimer. The images shown are stereo pairs, and the view is approximately perpendicular to the C3 axis. The Tl-1 and Tl-2 peaks are located at the sites corresponding to P0′ and P3′ sites shown in a. The relatively weaker densities of Tl-1 and Tl-2 peaks compared to those on surfaces indicate that the occupancy of Tl+ ions on these internal sites is much lower than on the surface ones. Note that only one monomer in the trimer contains a peak above 3 × σ level at P0′ site (Tl-1), suggesting that this site at the current conformational state is not yet fully accessible to Tl+/K+. Stereo pairs are shown in a–c. (TIF 7223 kb) [file 12915_2017_372_MOESM4_ESM.tif]

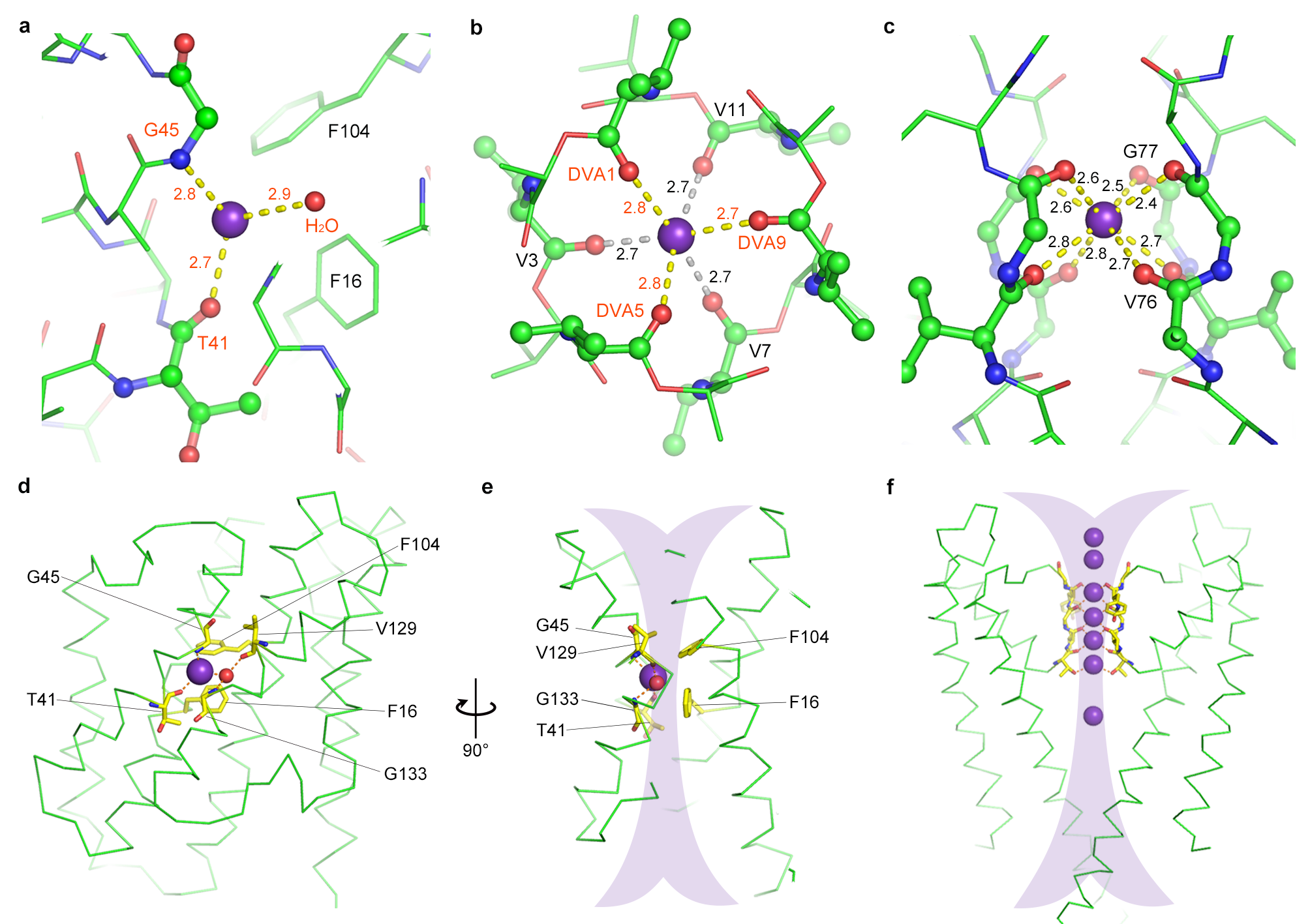

Supplement: Supplementary file 5 — Central putative monovalent cation-binding site and filter-like structure in SsTRIC compared to those of valinomycin and KcsA channel. a The ligands around the putative K+ (purple sphere, likely with partial occupancy and mixed with water molecules) at pore center of SsTRIC. Zoom-in view shows the trigonal coordination mode of K+; the side chain of Phe104 is located below K+. Dashed lines indicate the close interactions between K+ and its ligands with the bond length (Å) labeled nearby. The interaction between K+ and the backbone amide is not uncommon, as some similar interactions are found in the structure of gramicidin (PDB code 2IZQ, the K+-NH bond length is 3.1–3.4 Å, slightly weaker than the one we observed.). b The trigonal antiprism-type coordination of K+ in valinomycin (Cambridge Crystallographic Data Centre/CCDC accession code: VALINK). DVA and V indicate d- and l-valines, respectively. Gray dashed lines are the coordination bonds absent in SsTRIC. c The square antiprism-type coordination of K+ in KcsA. (PDB:1K4C). In panels a–c, K+ ions and water molecules are shown as purple and red spheres, respectively. The amino acid residues coordinating K+ ions are highlighted as ball-and-stick models. d and e The single layer filter-like structure sandwiched among M2 kink, M5 kink, Phe104, and Phe16 in SsTRIC. The putative K+ ion and a nearby water molecule are shown as sphere models. Amino acid residues (Phe16, Thr41, Gly45, Phe104, Val129, and Gly133) involved in binding K+ (or Na+) and water binding are shown as stick models. View in e is rotated ~90° with respect to view in a. f The canonical four-layer filter structure in KcsA channel. Purple funnels in e and f indicate the presumed permeation pathways for monovalent cations. (TIF 1214 kb) [file 12915_2017_372_MOESM5_ESM.tif]

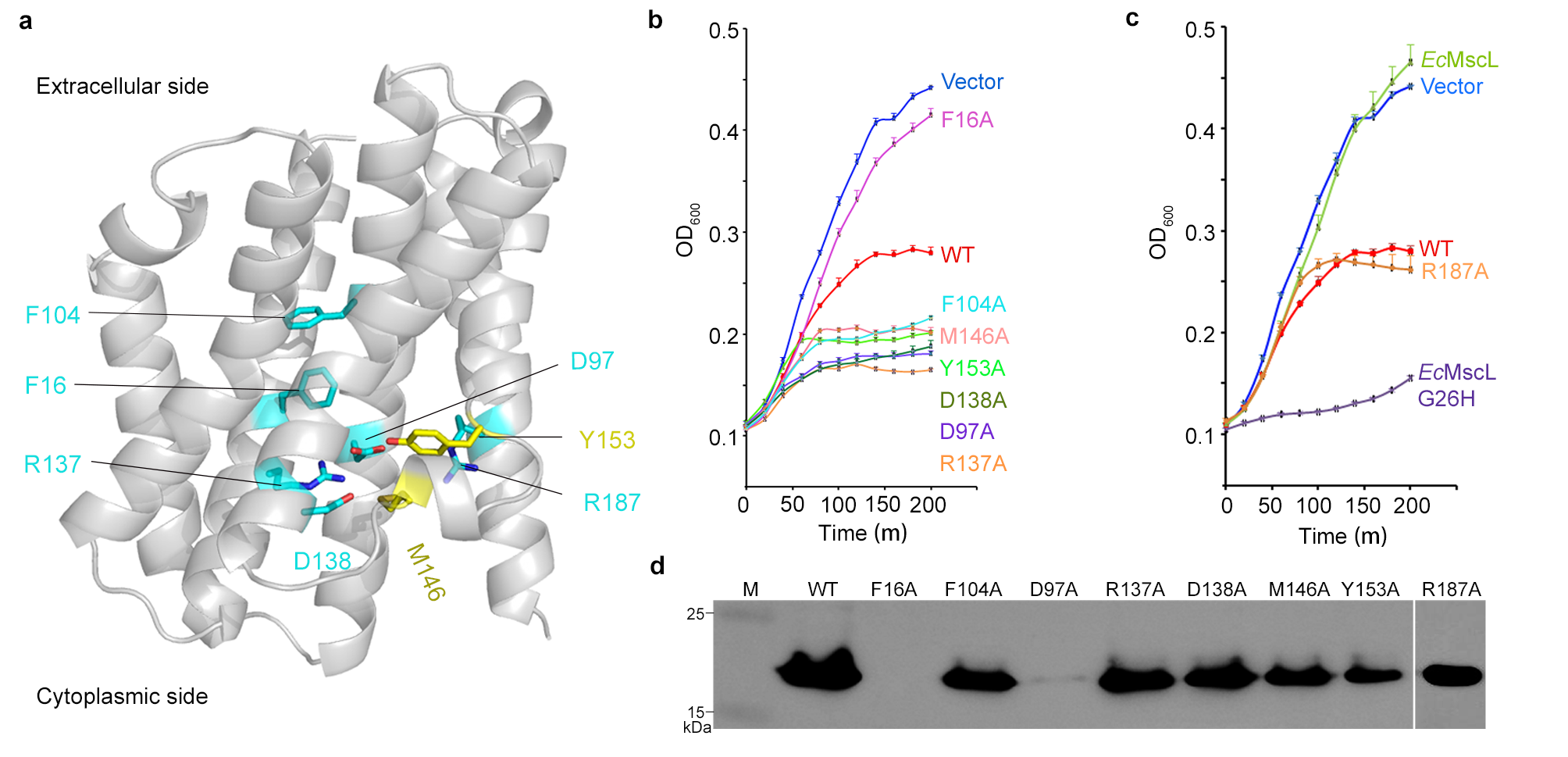

Supplement: Supplementary file 6 — In vivo assay on the function of SsTRIC and various mutants. a Side view of the SsTRIC monomer with the residues chosen for alanine-scanning mutagenesis highlighted in cyan or yellow. b Growth curves of E. coli cells expressing wild-type (WT) and various mutants of SsTRIC. c Control data for the cell growth assays. EcMscL: the large-conductance mechanosensitive channel (MscL) from E. coli. Overexpression of wild-type EcMscL does not inhibit the cell growth, as it remains tightly closed at the resting state, while its gain-of-function mutant G26H has a dramatic inhibitory effect on cell growth. They are included as controls for comparison with the phenotypes of SsTRIC and its mutants. Vector: empty pET21b vector used for expressing SsTRIC and mutants, included as a negative control; the R187A mutation site is relatively distant from the gating region and thus serves as an internal control. The error bars represent the standard errors of mean values (SEM, n = 3). d Western blots of the membrane fractions of E. coli cells expressing wild-type and mutant SsTRIC proteins. M molecular weight marker, WT wild-type SsTRIC. The protein was probed by anti-Histag antibody (see Methods for details). For each lane, 80 μg wet membrane solubilized by SDS-PAGE loading buffer was loaded. (TIF 632 kb) [file 12915_2017_372_MOESM6_ESM.tif]

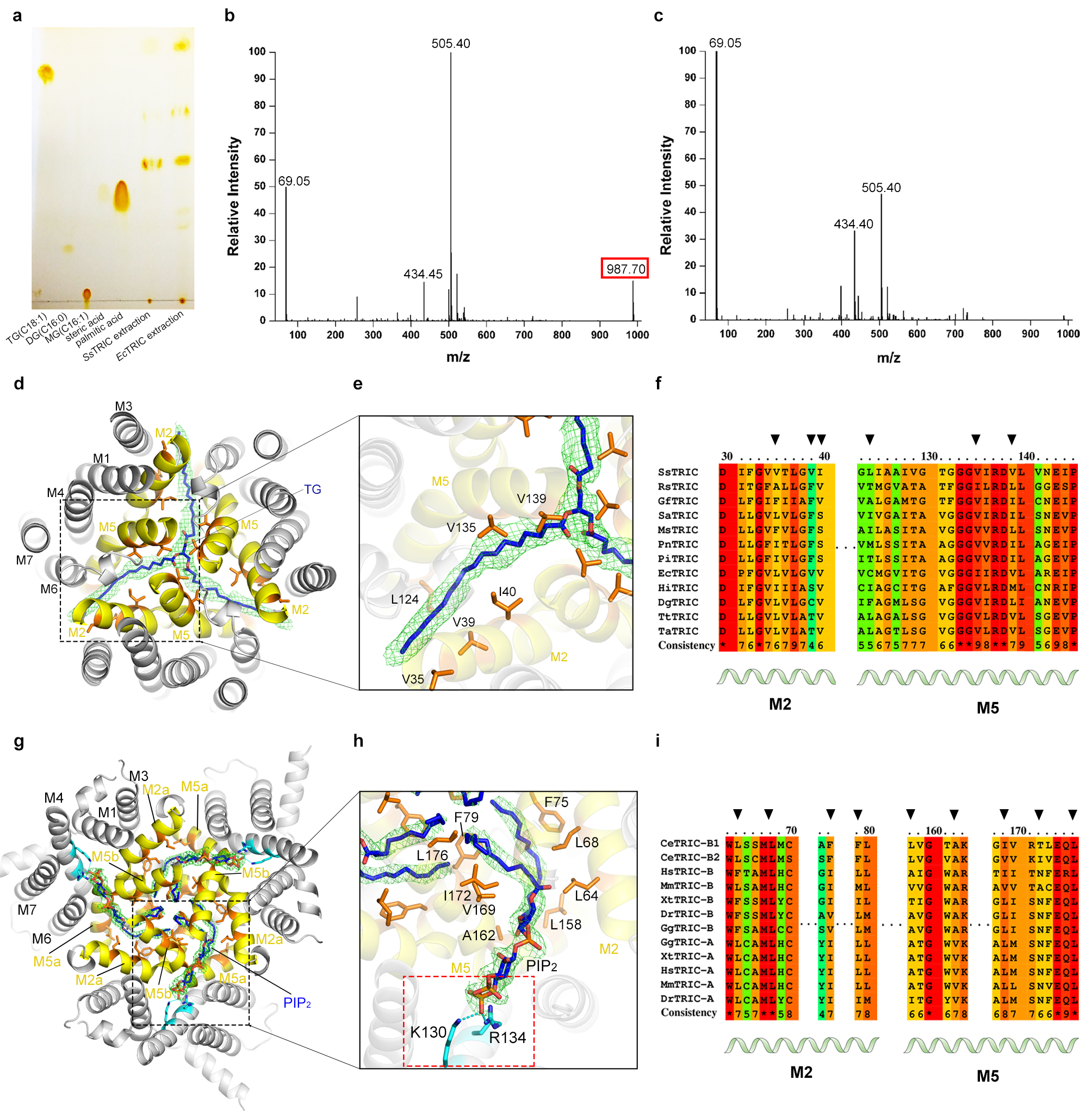

Supplement: Supplementary file 7 — Lipid molecules bound in the SsTRIC homotrimer. a Thin layer chromatography of the lipid samples extracted from purified SsTRIC and EcTRIC preparations. Three major neutral lipid bands from purified SsTRIC and EcTRIC samples are visible on the plate upon being stained by iodine. b Mass spectrometry analysis of lipid samples extracted from purified SsTRIC protein under electrospray ionization positive mode. Species with m/z at 987.70 may correspond to an ionized triacylglycerol molecule. c Mass spectrometry of the blank solution used for protein purification as a control. The species with m/z at 505.40 and 434.40 likely arise from the detergent. d Sectional view of SsTRIC trimer along the C3 axis from cytoplasmic side. Green meshes are 2F o-F c electron densities (contoured at +1.0 × σ level) potentially belonging to lipid cofactors. e Zoom-in view of a fatty acyl chain of TG molecule in SsTRIC. The amino acid residues involved in binding TG are shown as stick models. f Sequence alignment of the M2 and M5 regions in various prokaryotic TRIC members. Dark triangles indicate the amino acid residues involved in binding lipid molecules. Ss Sulfolobus solfataricus, Rs Rhodobacter sphaeroides, Gf Gramella forsetii, Sa Sulfolobus acidocaldarius, Ms Metallosphaera sedula, Pn Pyrobaculum neutrophilum, Pi Pyrobaculum islandicum, Ec Escherichia coli, Hi Haemophilus influenza, Dg Deinococcus geothermalis, Tt Thermus thermophilus, Ta Thermus aquaticus. g Sectional view of CeTRIC-B2 trimer (PDB:5EIK) along the C3 axis from cytoplasmic side. Green meshes are 2F o-F c electron densities of PIP2 molecules (contoured at +1.0 × σ level). h Zoom-in view of the region around PIP2 in CeTRIC-B2. i Sequence alignment of representative eukaryotic TRIC members in M2 and M5 regions involved in binding lipid acyl chains. Ce Caenorhabditis elegans, Hs Homo sapiens, Mm Mus musculus, Gg Gallus gallus, Dr Danio rerio, Xt Xenopus tropicalis. (TIF 2564 kb) [file 12915_2017_372_MOESM7_ESM.tif]

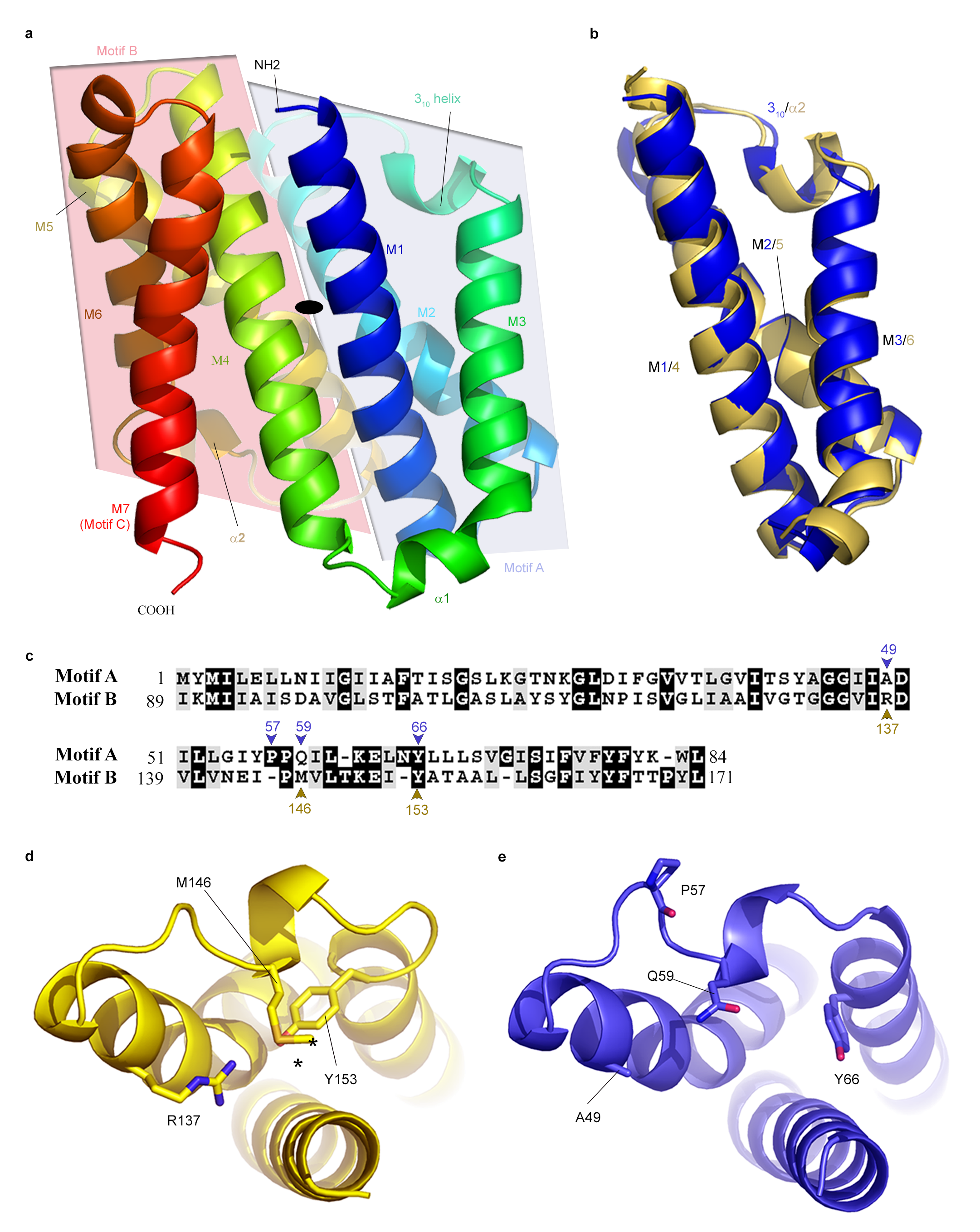

Supplement: Supplementary file 8 — Asymmetric features of the internal repeats in SsTRIC monomer. a Structure of an SsTRIC monomer represented as a cartoon ribbon model. Convex quadrilaterals in light blue and pink highlight the regions of motifs A and B, respectively. The dark solid ellipse at the center shows the position of a pseudo-C2 axis relating the two motifs. The view is along the pseudo-C2 axis running nearly parallel to the membrane plane. b Superposition of motif B (golden) with motif A (blue). c Alignment of the amino acid sequences of motifs A and B. Identical residues are in dark boxes; similar ones are in gray backgrounds. The blue and golden arrows highlight the key residues around the pore region. d The intracellular vestibule in motif B is occluded. e The extracellular vestibule of the pore harbored in motif A is open. The views are approximately along the pore axis. The key amino acid residues surrounding the pore region are shown as stick models. The asterisk symbols indicate the approximate location of the pore. (TIF 3075 kb) [file 12915_2017_372_MOESM8_ESM.tif]

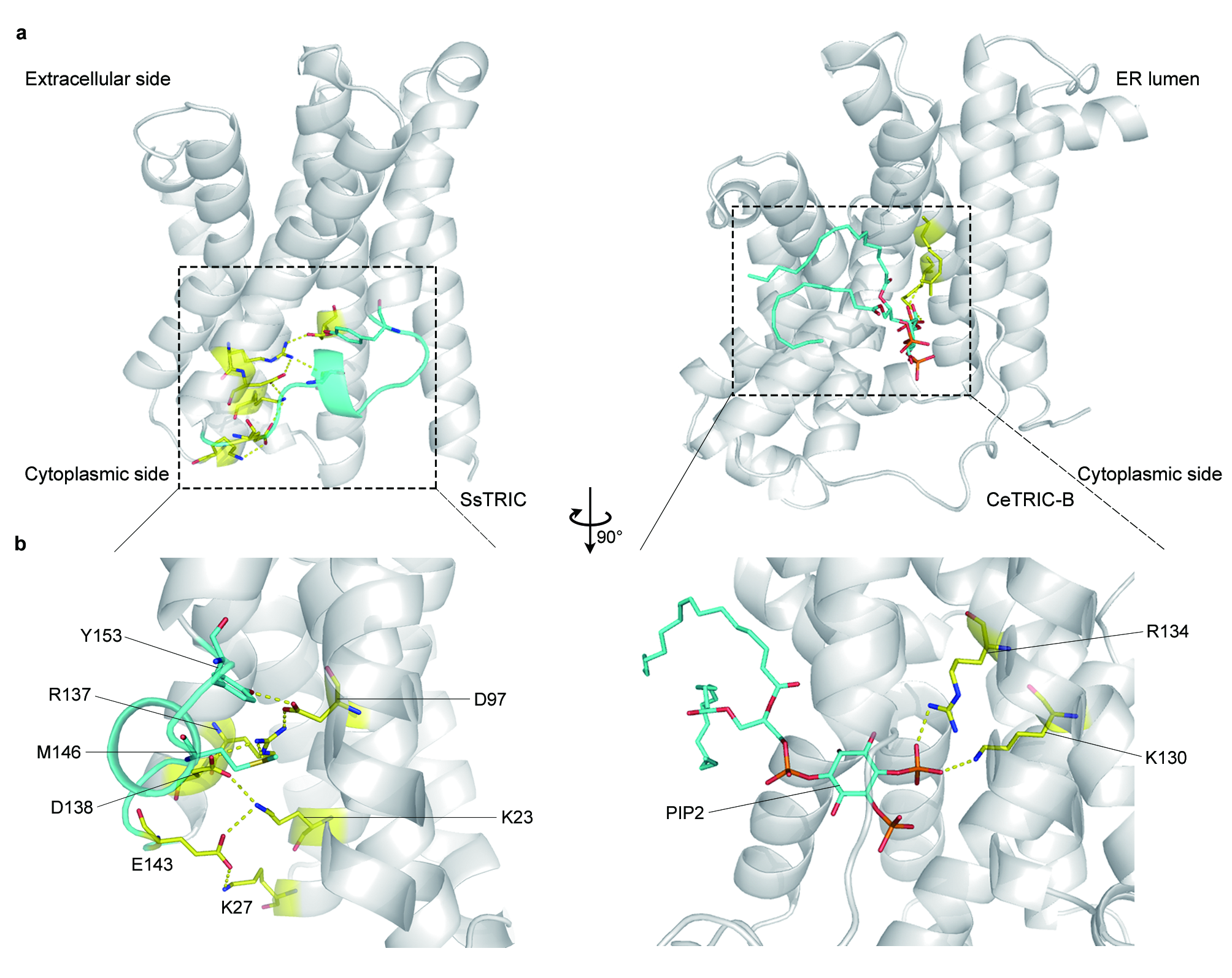

Supplement: Supplementary file 9 — Comparison between SsTRIC and CeTRIC-B2 structures. a Structures of SsTRIC and CeTRIC-B2 monomers superposed and viewed from the same angle. The SsTRIC (left) and CeTRIC-B2 (right, PDB code: 5EIK) backbones are represented as cartoons, and the lipid cofactor (PIP2) and key amino acid residues are shown as sticks. The plug motif/molecule is highlighted in cyan, while those involved in binding the plug are colored in yellow. b The position of plug motif in SsTRIC (left) compared to that of PIP2 in CeTRIC-B2 (right). The key residues involved in binding the plug motif/PIP2 are represented as yellow sticks. (TIF 2656 kb) [file 12915_2017_372_MOESM9_ESM.tif]

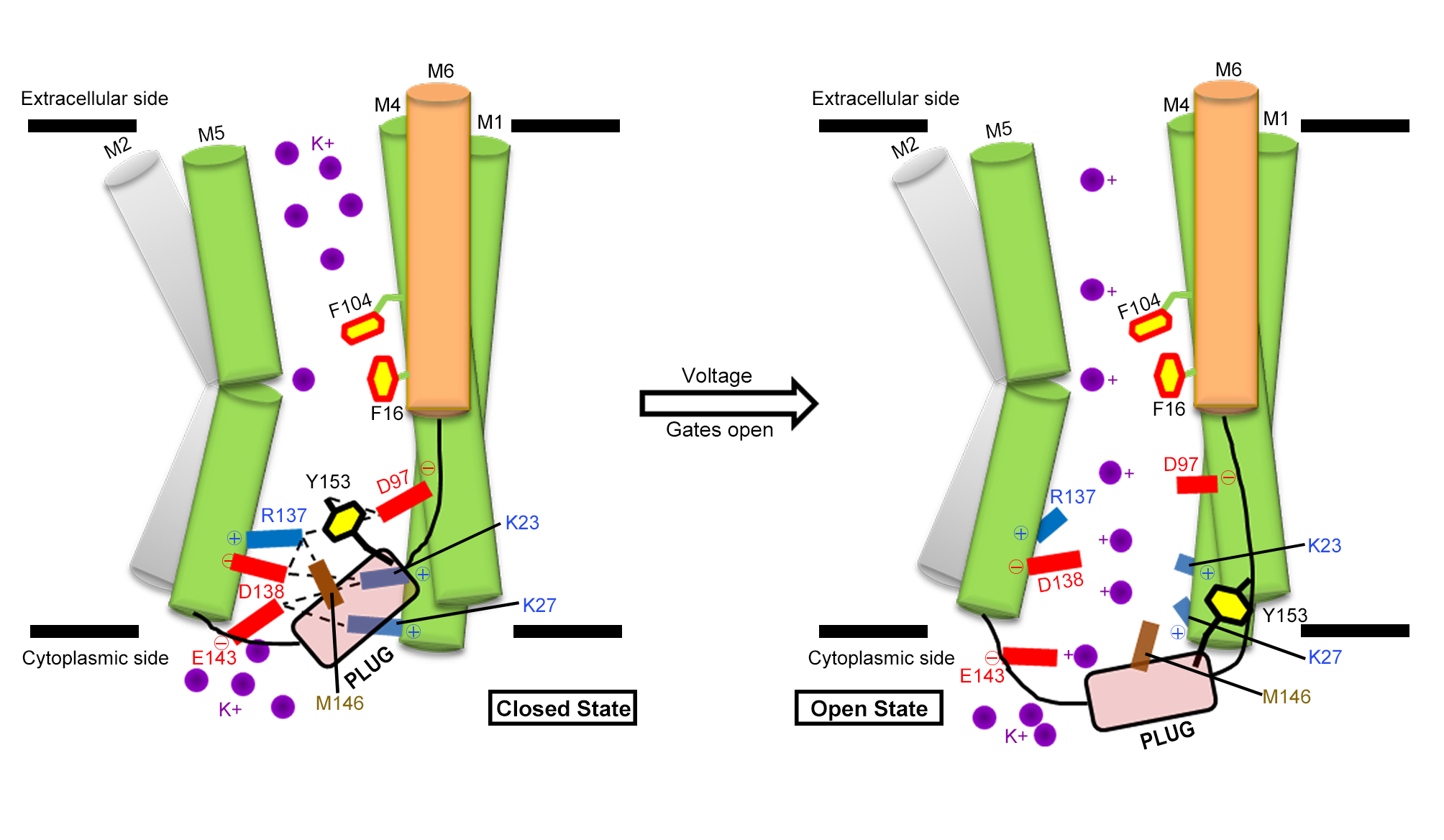

Supplement: Supplementary file 10 — Proposed model accounting for the gating mechanism of SsTRIC channel. At the closed state, the plug motif forms tight interactions with the pore lumen surface and blocks the cytoplasmic entrance. Upon activation by voltage, movement of the charged residues under electric field leads to destabilization of the interactions between plug motif and the potential voltage-sensing residues. The plug is thereby dislocated from the blocking position so that the cytoplasmic entrance of the hourglass-shaped pore is open for K+ ions to permeate through the pore. Blue and red sticks represent the positively and negatively charged residues, respectively; purple spheres represent K+ ions. For clarity, M3 and M7 helices are omitted in the model. For Kv or Nav channels, they generally adopt a string of positively charged amino acid residues (Arg and Lys) on a transmembrane helix (S4 helix) of the voltage-sensing domain (VSD) to sense electrical signals [51–53]. While SsTRIC channel does not contain a canonical VSD in its structure, it does have nine positively charged residues (Arg and Lys) distributed asymmetrically on two solvent-exposed surfaces (Fig. 4b), reflecting the positive-inside rule for membrane proteins [54]. The eight positively charged residues on the luminal side are not randomly distributed. Among them, six cluster around the cytoplasmic gate area, and three of them (Lys 27, Lys 23 on M1 helix and Arg137 on M5 helix) form an array of positively charged regions lining the interfacial groove between M1 and M5 (Fig. 4b). This array of positively charged residues is stabilized by three acidic residues: Asp97, Asp138, and Glu143 (Fig. 4c). Furthermore, three key residues from the plug motif interact directly or indirectly with these potential voltage-sensing residues (Fig. 4c). Hence, the Velcro-like structure may serve as a basis for the potential voltage-dependent regulation of SsTRIC channel activity. (TIF 743 kb) [file 12915_2017_372_MOESM10_ESM.tif]
